# Supplementary material for: Quality indicators for the primary prevention of cardiovascular disease in primary care: A systematic review
Source: PLoS One. 2024 Dec 5;19(12):e0312137. doi: 10.1371/journal.pone.0312137 (PMC11620663; doi:10.1371/journal.pone.0312137)
Supplement: S1 Table — (DOCX) [file pone.0312137.s001.docx]

**S1 Table.** Overview of search strategy

| Database | Search strategy |
| --- | --- |
| Ovid Medline | 1 (Cardiovasc* adj1 disease*).tw.  2 (heart adj1 disease*).tw.  3 myocardial infarction/  4 Cerebrovasc* event*.tw.  5 Cerebrovasc* disease*.tw.  6 Cerebrovasc* accident*.tw.  7 (Cardiac adj1 failure).tw.  8 Stroke*.tw. or stroke/  9 Transient isch*.tw.  10 TIA.tw.  11 (Haemorrhag* adj1 stroke).tw.  12 (hemorrhag* adj1 stroke).tw.  13 1 or 2 or 3 or 4 or 5 or 6 or 7 or 8 or 9 or 10 or 11 or 12  14 Risk*.tw.  15 Hypertension.tw.  16 (High adj2 pressure).tw.  17 Dyslipi*.tw.  18 Hyperlipi*.tw.  19 Smoking.tw.  20 atrial fibrillation.tw.  21 Diabetes.tw. or diabetes mellitus/  22 Alcohol.tw.  23 (Physical adj2 activity).tw.  24 Obesity.tw.  25 ((Mental adj1 health) or (mental adj1 disorder*) or depress* or anxiety or psychiat* or psycho* or well-being or (quality adj1 life) or (self adj1 esteem) or (self adj1 perception)).tw.  26 14 or 15 or 16 or 17 or 18 or 19 or 20 or 21 or 22 or 23 or 24 or 25  27 (primary adj2 prevention).tw.  28 (secondary adj2 prevention).tw.  29 (Prevention or control).tw.  30 27 or 28 or 29  31 (Quality adj1 care*).tw.  32 Quality assessment.tw.  33 Quality indicator*.tw.  34 Quality assurance.tw.  35 (medication* or medicine*).tw.  36 Quality improvement.tw.  37 Quality tool*.tw.  38 Quality monitor*.tw.  39 Quality metric.tw.  40 Quality criter*.tw.  41 Performance indicator.tw.  42 (Process adj2 care).tw.  43 Performance measure.tw.  44 Benchmark.tw. or benchmarking/  45 Outcome measure.tw.  46 Outcome indicator*.tw.  47 (Consensus or delphi).tw.  48 31 or 32 or 33 or 34 or 35 or 36 or 37 or 38 or 39 or 41 or 42 or 43 or 44 or 45 or 46 or 47  49 (Primary adj2 care).tw.  50 (General adj1 practi*).tw. or exp general practice/ or general practitioners/  51 Clinical practice.tw.  52 Family practice.tw.  53 (primary adj2 physician).tw.  54 healthcare delivery.tw.  55 49 or 50 or 51 or 52 or 53 or 54  56 13 and 26 and 30 and 48 and 55  57 limit 56 to (english language and yr="2012 -Current") |
| Ovid EMBASE | 1 (Cardiovasc* adj1 disease*).tw.  2 (heart adj1 disease*).tw.  3 myocardial infarction/  4 Cerebrovasc* event*.tw.  5 Cerebrovasc* disease*.tw.  6 Cerebrovasc* accident*.tw.  7 (Cardiac adj1 failure).tw.  8 Stroke*.tw. or stroke/  9 Transient isch*.tw.  10 TIA.tw.  11 (Haemorrhag* adj1 stroke).tw.  12 (hemorrhag* adj1 stroke).tw.  13 1 or 2 or 3 or 4 or 5 or 6 or 7 or 8 or 9 or 10 or 11 or 12  14 Risk*.tw.  15 Hypertension.tw.  16 (High adj2 pressure).tw.  17 Dyslipi*.tw.  18 Hyperlipi*.tw.  19 Smoking.tw.  20 atrial fibrillation.tw.  21 Diabetes.tw. or diabetes mellitus/  22 Alcohol.tw.  23 (Physical adj2 activity).tw.  24 Obesity.tw.  25 ((Mental adj1 health) or (mental adj1 disorder*) or depress* or anxiety or psychiat* or psycho* or well-being or (quality adj1 life) or (self adj1 esteem) or (self adj1 perception)).tw.  26 14 or 15 or 16 or 17 or 18 or 19 or 20 or 21 or 22 or 23 or 24 or 25  27 (primary adj2 prevention).tw.  28 (secondary adj2 prevention).tw.  29 (Prevention or control).tw.  30 27 or 28 or 29  31 (Quality adj1 care*).tw.  32 Quality assessment.tw.  33 Quality indicator*.tw.  34 Quality assurance.tw.  35 (medication* or medicine*).tw.  36 Quality improvement.tw.  37 Quality tool*.tw.  38 Quality monitor*.tw.  39 Quality metric.tw.  40 Quality criter*.tw.  41 Performance indicator.tw.  42 (Process adj2 care).tw.  43 Performance measure.tw.  44 Benchmark.tw. or benchmarking/  45 Outcome measure.tw.  46 Outcome indicator*.tw.  47 (Consensus or delphi).tw.  48 31 or 32 or 33 or 34 or 35 or 36 or 37 or 38 or 39 or 41 or 42 or 43 or 44 or 45 or 46 or 47  49 (Primary adj2 care).tw.  50 (General adj1 practi*).tw. or exp general practice/ or general practitioners/  51 Clinical practice.tw.  52 Family practice.tw.  53 (primary adj2 physician).tw.  54 healthcare delivery.tw.  55 49 or 50 or 51 or 52 or 53 or 54  56 13 and 26 and 30 and 48 and 55  57 limit 56 to (english language and yr="2012 -Current")  58 limit 57 to conference abstracts  59 57 not 58 |
| CINAHL Plus | (Cardiovasc* adj1 disease* OR heart adj1 disease* OR Stroke* OR Transient isch*or Haemorrhag* adj1 stroke OR hemorrhag* adj1 stroke OR TIA OR myocardial infarction OR Cerebrovasc* event* OR Cerebrovasc* disease* OR Cerebrovasc* accident* OR cardiac failure) AND (Risk* OR Hypertension OR High adj2 pressure OR Dyslipi* OR Hyperlipi* OR atrial adj1 fibrillation OR Smoking OR Diabetes OR diabetes mellitus OR Alcohol OR Physical adj2 activity OR Obesity OR Mental health OR mental disorder* OR depress* OR anxiety OR psychiat* OR well-being OR quality of life OR self esteem OR self perception) AND (Quality adj1 care* OR Quality assessment OR Quality indicator* OR Quality assurance OR medication* OR medicine* Quality improvement OR Quality tool* OR Quality monitor* OR Quality metric OR Quality criter* OR Performance indicator OR Process adj2 care OR Performance measure OR Benchmark OR benchmarking OR Outcome measure OR Outcome indicator* OR Consensus OR delphi) AND (Primary adj2 care OR General adj1 practi* OR general practice OR general practitioners OR Clinical practice OR Family practice OR primary adj2 physician OR healthcare delivery) AND (primary adj2 prevention OR secondary adj2 prevention OR prevention OR control) |
| SCOPUS | TITLE-ABS-KEY ("cardiovasc$ PRE/1 disease$" OR "heart PRE/1 disease$" OR "myocardial infarction" OR "cerebrovasc$ event$" OR "cerebrovasc$ disease$" OR "cerebrovasc$ accident$" OR "cardiac failure") OR ("stroke" OR "transient isch$" OR "haemorrhag$ PRE/1 stroke" OR "hemorrhag$ PRE/1 stroke") AND TITLE-ABS-KEY ("Risk$" OR "Hypertension" OR "High PRE/2 pressure" OR "Dyslipi$" OR "Hyperlipi$" OR "Smoking" OR "Diabetes" OR "diabetes mellitus" OR "Alcohol" OR "Physical PRE/2 activity" OR "Obesit$" OR "mental health" OR "mental disorder" OR "depress$" OR "anxiety" OR "psycho$" OR "psychiat$" OR "well-being" OR "quality of life" OR "self esteem" OR "self perception") AND TITLE-ABS-KEY ("Quality PRE/1 care$" or "Quality assessment" or "Quality indicator$" or "Quality assurance" or "medication$" or "medicine$" "Quality improvement" or "Quality tool$" or "Quality monitor$" or "Quality metric" or "Quality criter$" or "Performance indicator" or "Process PRE/2 care" or "Performance measure" or "Benchmark" or "benchmarking" or "Outcome measure" or "Outcome indicator$" or "Consensus" or "delphi") AND TITLE-ABS-KEY ("Primary PRE/2 care" or "General PRE/1 practi$" or "general practice" or "general practitioners" or "Clinical practice" or "Family practice" or "primary PRE/2 physician" or "healthcare delivery") AND TITLE-ABS-KEY ("primary PRE/2 prevention" OR "secondary PRE/2 prevention" OR "Prevention" OR "Control") AND ( EXCLUDE ( DOCTYPE,"cp" ) OR EXCLUDE ( DOCTYPE,"ed" ) ) AND ( LIMIT-TO ( PUBYEAR,2022) OR LIMIT-TO ( PUBYEAR,2021) OR LIMIT-TO ( PUBYEAR,2020) OR LIMIT-TO ( PUBYEAR,2019) OR LIMIT-TO ( PUBYEAR,2018) OR LIMIT-TO ( PUBYEAR,2017) OR LIMIT-TO ( PUBYEAR,2016) OR LIMIT-TO ( PUBYEAR,2015) OR LIMIT-TO ( PUBYEAR,2014) OR LIMIT-TO ( PUBYEAR,2013) OR LIMIT-TO ( PUBYEAR,2012) ) AND ( LIMIT-TO ( LANGUAGE,"English" ) ) |
