# Supplementary material for: Quality indicators for the primary prevention of cardiovascular disease in primary care: A systematic review
Source: PLoS One. 2024 Dec 5;19(12):e0312137. doi: 10.1371/journal.pone.0312137 (PMC11620663; doi:10.1371/journal.pone.0312137)
Supplement: S2 Table — (DOCX) [file pone.0312137.s002.docx]

**S2 Table.** Overview of grey literature search strategy: List of organisations and webpage reviewed

| **Organisations** | **Webpage** |
| --- | --- |
| Australian Council on Healthcare Standards | <https://www.achs.org.au/> |
| The Australian Commission on Safety and Quality in Health Care List | <https://www.safetyandquality.gov.au/standards/nsqhs-standards> |
| Australian Institute of Health and Welfare | <https://www.aihw.gov.au/> |
| Australian Government of Health and Ageing | <https://www.health.gov.au/> |
| Clinical Information Access Programme | <https://www.ciap.health.nsw.gov.au/> |
| National Institute of Clinical Studies | <https://catalogue.nla.gov.au/catalog/3625531> |
| Victorian Government Health Information | <https://www.health.vic.gov.au/> |
| Victorian Government Public Sector Residential Aged Care Services (PSRACS) quality Indicators | <https://www.health.vic.gov.au/residential-aged-care/quality-indicators-in-public-sector-residential-aged-care-services> |
| Improvement Foundation | <https://www.acnc.gov.au/charity/charities/721fd63e-38af-e811-a960-000d3ad24282/people> |
| Royal Australian College of General Practitioners | <https://www.racgp.org.au/> |
| Bettering the Evaluation and Care of Health (BEACH) studies | <https://www.sydney.edu.au/medicine-health/our-research/research-centres/bettering-the-evaluation-and-care-of-health.html> |
| Quality indicators. The Royal Australian College of General Practitioners: | <https://www.racgp.org.au/> |
| National Aged Care Mandatory Quality Indicator Program | <https://www.health.gov.au/our-work/qi-program> |
| Quality indicator app | <https://www.qpsbenchmarking.com/solutions/national-quality-indicator-app/> |
| Clinical Indicators | <https://www.racgp.org.au/running-a-practice/practicemanagement/general-practice-governance/clinical-indicators> |
| Care Quality Commission | <https://www.cqc.org.uk/> |
| CQUIN – Commissioning for Quality and Innovation Indicator | <https://www.england.nhs.uk/nhs-standard-contract/cquin/> |
| Department of Health | <https://www.gov.uk/government/organisations/department-of-health-and-social-care> |
| Dr. Foster Intelligence | <https://telstrahealth.co.uk/product/healthcare-intelligence-portal-quality/> |
| NHS Information Centre | <https://www.gov.uk/government/organisations/nhs-information-centre> |
| Clinical and Health Outcomes Knowledge Base | <https://digital.nhs.uk/article/1885/compendium-of-population-health-indicators> |
| NHS North West | <https://www.england.nhs.uk/north-west/> |
| National Institute for Health and Clinical Excellence (NICE) | <https://www.nice.org.uk/> |
| The Health Foundation | <https://www.health.org.uk/> |
| Stroke quality indicator | <https://archive.uhb.nhs.uk/stroke-quality-indicator.htm> |
| Accreditation Canada | <https://accreditation.ca/> |
| Canadian Institute for Health Information | <https://www.cihi.ca/en> |
| Health Canada | <https://www.canada.ca/en/health-canada.html> |
| Health Council of Canada | <https://www.healthcouncilcanada.ca/> |
| Public Health Agency of Canada | <https://www.canada.ca/en/public-health.html> |
