# Supplementary material for: Quality indicators for the primary prevention of cardiovascular disease in primary care: A systematic review
Source: PLoS One. 2024 Dec 5;19(12):e0312137. doi: 10.1371/journal.pone.0312137 (PMC11620663; doi:10.1371/journal.pone.0312137)
Supplement: S3 Table — (DOCX) [file pone.0312137.s003.docx]

## S3 Table. Full-text screening checklist

**Instruction:** The purpose of this checklist to guide full text screening and document reasons for exclusion of studies for final review. Read each criterion below and tick Yes (√) or No (☓) in the table provided

Covidence #: ____________

First author: ____________

Year: __________________

Screener initials: ________

Screening date: __/_/____(dd/mm/yy)

| **SN** | **Criteria** | **Yes** | **No** | **NA** | **Remarks** |
| --- | --- | --- | --- | --- | --- |
| 1. | Related to stroke or cardiovascular diseases |  |  |  |  |
| 2. | Focussed on quality indicators (process or structural indicators) |  |  |  |  |
| 3. | Defined indicators |  |  |  |  |
| 4. | Provided detailed information on calculation of quality indicators |  |  |  |  |
| 5. | Includes primary care or general practitioners setting |  |  |  |  |
| 6. | Consensus statement or guideline |  |  |  |  |
| 7. | Ineligible study design (e.g. case report, case series, letter to editor, systematic review) |  |  |  |  |
| 8. | Availability of full text |  |  |  |  |
| 9 | Duplicate study (missed at earlier stage) |  |  |  |  |
| 10. | Other (Please specify) …………………………………. |  |  |  |  |

**Overall comment**

|  |
| --- |

**Final decision**

Include

Exclude
