# Supplementary material for: Quality indicators for the primary prevention of cardiovascular disease in primary care: A systematic review
Source: PLoS One. 2024 Dec 5;19(12):e0312137. doi: 10.1371/journal.pone.0312137 (PMC11620663; doi:10.1371/journal.pone.0312137)
Supplement: S4 Table — (DOCX) [file pone.0312137.s004.docx]

## S4 Table. Study characteristics extraction table template

| **Author** | **Year** | **Country** | **World Bank category^a^** | **Study design/Methodology^b^** | **Study period** |
| --- | --- | --- | --- | --- | --- |
|  |  |  |  |  |  |
|  |  |  |  |  |  |
|  |  |  |  |  |  |
|  |  |  |  |  |  |
| **^a^**World bank category include low, lower-middle, upper-middle, and high income; **^b^**study design/methodology include cross sectional study, cohort study, randomised controlled trial, quality improvement study, mixed methods, pre-post study, development and validation, grey literature, review and (modified) Delphi method. | | | | | |
