# Supplementary material for: Quality indicators for the primary prevention of cardiovascular disease in primary care: A systematic review
Source: PLoS One. 2024 Dec 5;19(12):e0312137. doi: 10.1371/journal.pone.0312137 (PMC11620663; doi:10.1371/journal.pone.0312137)
Supplement: S5 Table — (DOCX) [file pone.0312137.s005.docx]

## S5 Table. Quality indicators data extraction table template

| **Author (Year)** | **Country** | **Condition^a^** | **Themes^b^** | **Domains^c^** | **Type of Indicator^d^** | **Age group (years)** | **Numerator** | **Denominator** | **Frequency of collection^e^** |
| --- | --- | --- | --- | --- | --- | --- | --- | --- | --- |
|  |  |  |  |  |  |  |  |  |  |
|  |  |  |  |  |  |  |  |  |  |
|  |  |  |  |  |  |  |  |  |  |
|  |  |  |  |  |  |  |  |  |  |
|  |  |  |  |  |  |  |  |  |  |
| **^a^**Condition was based on denominator include no existing risk factor, at risk of cardiovascular disease, atrial fibrillation, hypertension, diabetes, kidney disease, mental health, dyslipidaemia, transient ischaemic attack, smoking, heart failure and others; **^b^**Themes were based on the numerator; **^c^**domains include organisation of care, clinical processes, lifestyle management, referral, and attainment of risk factor targets; **^d^**Type of indicator include structure, process, and outcome; **^e^**frequency of collection based on the definition of indicator. | | | | | | | | | |
