# Supplementary material for: Quality indicators for the primary prevention of cardiovascular disease in primary care: A systematic review
Source: PLoS One. 2024 Dec 5;19(12):e0312137. doi: 10.1371/journal.pone.0312137 (PMC11620663; doi:10.1371/journal.pone.0312137)
Supplement: S6 Table — (DOCX) [file pone.0312137.s006.docx]

## S6 Table. Appraisal of Indicators through Research and Evaluation (AIRE) instrument criteria to evaluate quality indicators

| **S.N.** | **AIRE Criteria** | **Score** |
| --- | --- | --- |
| **1.** | **Purpose, relevance and organizational context** |  |
| i. | The purpose of the indicator is described clearly. |  |
| ii. | The criteria for selecting the topic of the indicator are described in detail. |  |
| iii. | The organizational context of the indicator is described in detail. |  |
| iv. | The quality domain the indicator addresses is described in detail. |  |
| v. | The health care process covered by the indicator is described and defined in detail. |  |
|  | Sub-Total |  |
|  | % |  |
| **2** | **Stakeholder involvement** |  |
| i. | The group developing the indicator includes individuals from all relevant professional groups. |  |
| ii. | Considering the purpose of the indicator, all relevant stakeholders have been involved at some stage of the development process. |  |
| iii. | The indicator has been formally endorsed. |  |
|  | Sub-Total |  |
|  | % |  |
| **3** | **Scientific evidence** |  |
| i. | Systematic methods were used to search for scientific evidence. |  |
| ii. | The indicator is based on recommendations from an evidence-based guideline or studies published in peer-reviewed scientific journals |  |
| iii. | The supporting evidence has been critically appraised. |  |
|  | Sub-Total |  |
|  | % |  |
| **4** | **Additional evidence, formulation, usage** |  |
| i. | The numerator and denominator are described in detail. |  |
| ii. | The target patient population of the indicator is defined clearly. |  |
| iii. | A strategy for risk adjustment has been considered and described. |  |
| iv. | The indicator measures what it is intended to measure (validity). |  |
| v. | The indicator measures accurately and consistently (reliability). |  |
| vi. | The indicator has sufficient discriminative power. |  |
| vii. | The indicator has been piloted in practice. |  |
| viii. | The efforts needed for data collection have been considered. |  |
| ix. | Specific instructions for presenting and interpreting results. |  |
|  | Sub-Total |  |
|  | % |  |
|  | **Grand Total** |  |
|  | % |  |

**Scoring criteria**

| **Scoring (1 to 4 )** | **Criteria** |
| --- | --- |
| 1—strongly disagree | Confident that the criterion has not been fulfilled or no information was available |
| 2/3—disagree/agree | Unsure whether the criterion has been fulfilled |
| 4—strongly agree | Confident that the criterion has been fulfilled |
