# Supplementary material for: Quality indicators for the primary prevention of cardiovascular disease in primary care: A systematic review
Source: PLoS One. 2024 Dec 5;19(12):e0312137. doi: 10.1371/journal.pone.0312137 (PMC11620663; doi:10.1371/journal.pone.0312137)
Supplement: S9 Table — (DOCX) [file pone.0312137.s009.docx]

**S9 Table.** List of unique quality indicators by conditions

| **Numerator** | **Denominator** | **Age group (years)** | **Frequency** |
| --- | --- | --- | --- |
| **No existing RF (n=114)** | | | |
| Screened for tobacco use one or more times and who received cessation counselling intervention if identified as a tobacco user. | All patients | ≥18 | Every 2 years |
| Access to 12-leadECG, ambulatory ECG Holter monitoring, transthoracic echocardiogram, and CT calcium scoring. | All healthcare centres or units | NA | Not specified |
| Dedicated multi-disciplinary team | All healthcare centres or units | NA | Not specified |
| Participate in a registry or common database to record patients’ BMI, BP, LDL-C, HbA1c, and renal function | All healthcare centres or units | NA | Not specified |
| Had cardiovascular risk assessment documented | All indigenous patients registered for CVD services | ≥20 | Every 2 years |
| Number of regular clients who had a BMI classified as underweight, healthy, overweight or obese | All regular clients who had a BMI recorded. | >15 | Every 12 months |
| Number of regular clients aged who had their BMI recorded | All regular clients | >15 | Every 12 months |
| Number of regular clients who have had all CVD risk factors recorded (smoking status, diabetes, systolic blood pressure, total cholesterol, HDL cholesterol, age and sex) | All regular clients | 45-74 | Every 2 years |
| Recording of regular clients who smoking status: current smoker, ex-smoker or never smoked | All regular clients | >15 | Every 12 months |
| The number of regular clients who have had their alcohol consumption recorded | All regular clients | >15 | Every 2 years |
| The number of regular clients who are immunised against influenza | All regular clients | ≥65 | Every 12 months |
| Record of absolute cardiovascular disease risk | All patients | 45-69 | Not specified |
| Record of alcohol use | All patients | 40-69 | Not specified |
| Record of BMI | All patients | 40-69 | Not specified |
| Record of diet | All patients | 40-69 | Not specified |
| Record of fasting blood glucose | All patients | 45-69 | Not specified |
| Record of lipids (total cholesterol, high-density lipoprotein, low-density lipoprotein, triglycerides) | All patients | 45-69 | Not specified |
| Record of physical activity | All patients | 40-69 | Not specified |
| Record of smoking status (current) | All patients | 40-69 | Not specified |
| Record of systolic and diastolic BP | All patients | 40-69 | Not specified |
| Record of waist circumference | All patients | 40-69 | Not specified |
| Screened for atrial fibrillation by pulse palpation or electrocardiogram in clinical practice who were not already being monitored for atrial fibrillation | All patients | ≥65 | Every 2 weeks |
| Screened for BP in clinical practice who were not already being monitored for hypertension | All patients | ≥40 | Every 2 weeks |
| Record of BP control | All patients | All | Not specified |
| Record of cholesterol management | All patients | All | Not specified |
| Record of smoking cessation | All patients | All | Not specified |
| The number of days until the GP’s 3rd available appointment. | All patients | NA | Not specified |
| Number of patients with a care plan documented | All patients | All | Not specified |
| Previously diagnosed with or currently have an active diagnosis of clinical atherosclerotic cardiovascular disease | All patients | ≥18 | Not specified |
| Screened for tobacco use ≥1 time and who received cessation counselling intervention if identified as a tobacco user | All patients | ≥18 | Every 2 years |
| ≥1 team meeting about quality improvement relating to CVD | All practices | NA | Not specified |
| An up-to-date directory of prevention activities/organisations available locally (e.g. gyms, walking groups, weight-watchers etc.) | All practices | NA | Not specified |
| Computer used for creating medication prescriptions | All practices | NA | Not specified |
| Critical incident register | All practices | NA | Not specified |
| CVD risk advice (e.g. about modifiable risk factors such as diet and exercise) integrated with the patient medical record system | All practices | NA | Not specified |
| CVD risk assessment tool integrated with the patient medical record system (e.g. so that the CVD event risk score is entered directly in to the patient’s medical record) | All practices | NA | Not specified |
| Doctors have direct access to medical guidelines (either on paper or electronic) in their treatment rooms | All practices | NA | Not specified |
| Nurses attend ≥1 training/continuing medical education event on CVD | All practices | NA | Every 5 years |
| Nurses take part in local/community campaigns or actions on CVD risk prevention (e.g. stop smoking campaigns, fun-runs etc.) | All practices | NA | Not specified |
| Offer regularly two or many consultations to provide advice on patients’ life style | All practices | NA | Not specified |
| Participate in public health care programmes on life style (physical exercise, stop smoking) | All practices | NA | Not specified |
| Procedure for the management of patient information in relation to detailed examination results and the documentation of measures that were taken (e.g., blood examinations) | All practices | NA | Not specified |
| Procedure for the management of patient information in relation to the review of detailed examination results by the doctor (in terms of outgoing needs) | All practices | NA | Not specified |
| Produce a quality report | All practices | NA | Not specified |
| Record in the electronic or paper-based patient record that the CVD standardized risk assessment tool has been offered | All practices | NA | Not specified |
| Undertaken ≥1 clinical audit | All practices | NA | Every 12 months |
| Use a computer-supported patient file system | All practices | NA | Not specified |
| Use a system for recalling populations at risk for preventive care regarding CVD | All practices | NA | Not specified |
| Use a system for recalling populations at risk for preventive care regarding influenza | All practices | NA | Not specified |
| Use case finding methods to detect patients with cardiovascular risk factors | All practices | NA | Not specified |
| Use CVD standardized risk assessment tool | All practices | NA | Not specified |
| Patients with a blood pressure measurement recorded | All patients | ≥40 | Every 5 years |
| Controlling high BP | All patients | All | Every 12 months |
| Screening and smoking cessation intervention | All patients | All | Every 12 months |
| Diagnosis of CVD should be clearly identifiable from the paper and/or electronic record | All practices | NA | Not applicable |
| Information leaflet about CVD should be available at the practice for patients to take home or read in the practice | All practices | NA | Not applicable |
| Medical record should contain a detail of current prescribed medications | All practices | NA | Not applicable |
| Medical record should contain a summary list of major medical problems | All practices | NA | Not applicable |
| Medical record should contain information about intolerances and contradictions to medications | All practices | NA | Not applicable |
| Record of ≥1 GP per practice should attend ≥1 training/continuing medical education event on CVD | All GPs | NA | Every 12 months |
| Record of ≥1 Nurse per practice should attend ≥1 training/continuing medical education event on CVD | All Nurses | NA | Every 12 months |
| Record of ≥1 training/continuing medical education event on CVD by GPs | All GPs | NA | Every 5 years |
| Record of ≥1 training/continuing medical education event on CVD by Nurses | All Nurses | NA | Every 5 years |
| ≥1 level of physical activity/exercise capacity | All patients | ≥16 | Every 5 years |
| CVD risk assessment including risk factors and risk profiling | All patients | All | Not specified |
| Offered risk assessment using a standardised tool | All patients | ≥40 | Not specified |
| Record of ≥1 smoking | All patients | ≥16 | Not specified |
| Record of ≥1 weight or BMI | All patients | 16-55 | Every 5 years |
| Record of ≥1 BP measurement | All patients | >16 | Every 5 years |
| Record of ≥1 cholesterol | All patients | >35 | Every 5 years |
| Record of ≥1 waist circumference | All patients | ≥16 | Every 5 years |
| Record of ≥1 weight or BMI | All patients | ≥55 | Every 12 months |
| Record of their physical activity/exercise capacity | All patients | ≥16 | Every 5 years |
| Number of Aboriginal and Torres Strait Islander patients aged > 35 years screened using both eGFR and urinary albumin in the past 24 months. | Number of Aboriginal and Torres Strait Islander patients aged > 35 years seen in the past 24 months | >35 | Every 2 years |
| Recording of alcohol status (drinker or non-drinker) status | Active patients aged 15-80 years | 15-80 | Not specified |
| Recording of smoking status (current smoker, ex-smokers, or never smoked) status | Active patients aged 10-80 years | 10-80 | 2 years |
| Risk variables such as age, gender, smoking status, total and HD cholesterol level and blood pressure collected | Active patients aged 45-74 years | 45-74 | 2-5 years |
| Risk variables such as age, gender, smoking status, total and HD cholesterol level and blood pressure collected | Active patients aged 35-74 years who identify as Aboriginal or Torress Strait Islander | 35-74 | 2-5 years |
| Record of ≥2 encounters in 12 months (or 1 or more preventive encounters in past 12 months) and screened about tobacco use ≥1 within 24 months and who received cessation counselling intervention if identified as a tobacco user | All patients | ≥18 | Every 12 months |
| Current smokers who have a record of an offer of support and treatment within the preceding 24 months | All patients aged >15 years | >15 | Every 2 years |
| Maintains a register of patients aged 18 years or over with a BMI ≥30 in the preceding 12 months | All practices | > 18 | Every 12 months |
| Record of blood pressure | All patients aged ≥45 years | ≥45 | Every 5 years |
| Recording of patients who had a blood test for total cholesterol where total cholesterol value was 5.2mmol/L | All patients | ≥20 | Not specified |
| Recording of patients who had a full fasting lipid profile measured | All patients | ≥40 (Men) ≥50 (Women) | Every 5 years |
| Recording of patients who had a measured or self-reported BMI of 30 kg/m^2^ | All patients | ≥20 | Not specified |
| Recording of patients who had their height and weight measured by a healthcare professional | All patients | ≥20 | Not specified |
| Recording of patients who have a diagnosis of diabetes (Type I or Type II) based on a validated administrative data algorithm, recorded primary healthcare provider diagnosis in the EMR, or self-report | All patients | ≥20 | Not specified |
| Recording of patients who have a diagnosis of hypertension OR elevated BP (140mmHg systolic or 90mmHg diastolic) OR who self-report having hypertension | All patients | ≥20 | Not specified |
| Recording of patients who have had a fasting blood glucose screening or HbA1c screening | All patients | ≥40 | Every 3 years |
| Recording of patients who have had their BP measured by a healthcare professional | All patients | ≥20 | Not specified |
| Recording of patients with most recent blood test showing LDL-C >2 mmol/L | All patients | ≥20 | Not specified |
| Recording of self-reported being/were identified as a current smoker, either daily or occasionally | All patients | ≥20 | Not specified |
| Appropriate CVD risk factor screening: ≥1 recorded smoking status and systolic BP and total and high-density lipoprotein cholesterol levels | All patients | ≥45 | Every 2 years |
| Appropriate CVD risk factor screening: ≥1 recorded smoking status and systolic BP and total and high-density lipoprotein cholesterol levels | All Aboriginal and Torres Strait Islander Australian patients | ≥35 | Every 2 years |
| Record of high CVD risk, or a calculated 5-year CVD risk exceeding 15%, as assessed with the Framingham equation. | All patients | ≥18 | Not specified |
| Ankle brachial index performed | All patients | All | Not specified |
| Appointment of ambulatory BP monitoring performed | All patients | All | Not specified |
| Daily proteinuria performed | All patients | All | Not specified |
| Doppler carotid arteries performed | All patients | All | Not specified |
| Glucose tolerance test performed | All patients | All | Not specified |
| Holding Echo-cardioscopy was conducted | All patients | All | Not specified |
| Performed electrolyte content in plasma | All patients | All | Not specified |
| Performing of ECG | All patients | All | Not specified |
| Pulse wave velocity performed | All patients | All | Not specified |
| Record of anamnesis taken | All patients | All | Not specified |
| Record of blood level glucose was conducted | All patients | All | Not specified |
| Record of conducted BP at the legs | All patients | All | Not specified |
| Record of conducted BP in both arms | All patients | All | Not specified |
| Record of ophthalmoscopy performed | All patients | All | Not specified |
| Record of physical examination | All patients | All | Not specified |
| Renal ultrasonography performed | All patients | All | Not specified |
| Research creatinine content in plasma was conducted | All patients | All | Not specified |
| Research of LDL | All patients | All | Not specified |
| Research of triglycerides | All patients | All | Not specified |
| Record of cholesterol | All patients | All | Not specified |
| **At risk of CVD (n=184)** | | | |
| Documented discussion with a member of the multidisciplinary team about their treatment goals, preference, and values | All patients at high risk of CVD | ≥18 | Every 12 months |
| Patients who self-identify as non-smokers. | Patients with established or high risk for ASCVD who previously self-identified as smokers. | ≥18 | Not specified |
| Record of baseline LDL-C ≥1.8 mmol/L (≥70 mg/dL) who are prescribed lipid lowering therapy. | All patients with very high risk for ASCVD and a baseline LDL-C ≥ 1.8 mmol/L (≥70 mg/dL) | ≥18 | Not specified |
| Record of follow up to assess and address cardiovascular risk factors | All patients with established or high risk for ASCVD | ≥18 | Every 12 months |
| Record of LDL-C levels at or below that recommended for their estimated cardiovascular risk. | All patients with established or high risk for ASCVD | ≥18 | Not specified |
| Satisfaction about risk factor control captured | All patients at high risk of CVD | NA | Every 12 months |
| Record of pulse check (or ECG) to identify rhythm | Number of people ≥65 years of age with risk factors for AF | ≥65 | Not specified |
| Record of achieved LDL-C≤2 mmol/L | All patients with high risk of CVD | ≥18 | Not specified |
| Record of BMI | All patients with high risk of CVD | ≥18 | Not specified |
| Record of most recent smoking status | All patients with high risk of CVD | ≥18 | Not specified |
| BP ≤ 140/90 mmHg in last measurement | All patients at risk of stroke | All | Every 12 months |
| Referred to computed tomography (CT) or nuclear magnetic resonance (NMR) | All new patients with high risk of stroke | ≥18 | Not specified |
| Total cholesterol ≤ 5mmol in last measurement | All patients at risk of stroke | All | Every 12 months |
| Achieved treatment targets for BP (<130 mmHg), total cholesterol (<4.0 mmol/L) and smoking cessation | All patients assessed for modifiable risk factors | ≥18 | Not specified |
| Exceeded treatment targets for BP and total cholesterol and not prescribed appropriate medications | All patients assessed for modifiable risk factors | ≥18 | Not specified |
| Exceeded treatment targets for BP and total cholesterol despite being prescribed appropriate medications | All patients assessed for modifiable risk factors | ≥18 | Not specified |
| Prescribed BP and lipid lowering drugs respectively | All patients at high risk of CVD | ≥18 | Not specified |
| Record of modifiable risk factors (smoking, BP, total cholesterol) assessed | All patients at high risk of CVD | ≥18 | Every 12 months |
| Aspirin use | All patients at risk of CVD | 18-85 | Not specified |
| Record of calculated WHO/ISH <30 % or/and history of CVD whose most recent BP measurement was at normal range (< 140/90 mmHg) | All patients at high risk of CVD | All | Not specified |
| Record of calculated WHO/ISH ≥ 30 % or/and history of CVD prescribed aspirin | All patients at high risk of CVD | ≥18 | Not specified |
| Record of calculated WHO/ISH ≥ 30 % or/and history of CVD prescribed statin | All patients at high risk of CVD | ≥18 | Not specified |
| Record of calculated WHO/ISH ≥ 30 % or/and history of CVD prescribed triple therapy (station, aspirin, and BP medication) | All patients at high risk of CVD | ≥18 | Not specified |
| Record of calculated WHO/ISH ≥ 30 % or/and history of CVD whose most recent BP measurement was at normal range (< 140/90 mmHg) | All patients at high risk of CVD | ≥18 | Not specified |
| Record of blood lipid levels i) TC (at risk, > 4 mmol/L); ii) high-density lipoprotein cholesterol (at risk, < 1 mmol/L); iii) LDL cholesterol (at risk, > 2 mmol/L); & iv) triglycerides (at risk, > 1.5 mmol/L) | All patients at risk of CVD | 40-64 | Every 12 months |
| Record of physical activity level, which combined assessment of duration of vigorous and moderate physical activity (score range, 0–8; at risk,  < 4). | All patients at risk of CVD | 40-64 | Every 12 months |
| Record of a history of gestational diabetes mellitus, or impaired glucose tolerance or impaired fasting glycaemia | All patients at risk of CVD | 40-64 | Every 12 months |
| Record of alcohol consumption (at risk, > 2 standard drinks per day) | All patients at risk of CVD | 40-64 | Every 12 months |
| Record of BMI (at risk, 25 kg/m^2^) | All patients at risk of CVD | ≥40 | Every 12 months |
| Record of BP (at risk, > 135/85 mmHg) | All patients at risk of CVD | 40-64 | Every 12 months |
| Record of current smoking status | All patients at risk of CVD | 40-64 | Every 12 months |
| Record of fasting blood sugar (at risk, 5.5–6.9 mmol/L) | All patients at risk of CVD | 40-64 | Every 12 months |
| Record of oral glucose tolerance test was recommended for 5.5–6.9 mmol/L, at risk patients | All patients at high risk of CVD | 40-64 | Every 12 months |
| Record of serves of fruit and vegetables per day (at risk, < 7 serves per day) | All patients at risk of CVD | 40-64 | Every 12 months |
| Record of waist circumference > 102 cm in males or > 88 cm in females | All patients at risk of CVD | 40-64 | Every 12 months |
| Number of persons aged ≥65 years with >5 different medication prescriptions (within one quarter) per year | Number of persons aged ≥65 years with ≥1 drug prescription | ≥65 | Every 12 months |
| Record of influenza vaccination | Number of persons aged ≥65 years | ≥65 | Every 12 months |
| Blood glucose diary completed | All patients at high risk of CVD | ≥18 | Not specified |
| Blood glucose or HbA1c measurements as recommended in the guidelines | All patients at high risk of CVD | ≥18 | Not specified |
| BP diary completed | All patients at high risk of CVD | ≥18 | Not specified |
| BP measurements as recommended in guidelines | All patients at high risk of CVD | ≥18 | 1 - 12 months |
| BP target(s) achieved | All patients at high risk of CVD | ≥18 | Not specified |
| Change in BP medication by a clinician if BP >140/90 or >130/80 and diabetes or kidney disease | All patients at high risk of CVD | ≥18 | Not specified |
| Change in BP treatment plan (Medication or lifestyle changes and/or re-assessment plan) if BP >140/90 or >130/80 and diabetes or kidney disease | All patients at high risk of CVD | ≥18 | Not specified |
| Change in diabetes medication by a clinician if HbA1c >7% or fasting plasma glucose >7 mmol/L | All patients at high risk of CVD | ≥18 | Not specified |
| Change in diabetes treatment plan if HbA1c >7% or fasting plasma glucose >7 mmol/L | All patients at high risk of CVD | ≥18 | Not specified |
| Change in lipid-lowering medication by a clinician if LDL-C ≥2 mmol/L or apo B ≥0.8 g/L | All patients at high risk of CVD | ≥18 | Not specified |
| Change in lipid-lowering treatment plan if LDL-C ≥2 mmol/L or apo B ≥0.8 g/L | All patients at high risk of CVD | ≥18 | Not specified |
| Education on self-management for diabetes | All patients at high risk of CVD | ≥18 | Not specified |
| Education on self-management for diabetes received | All patients at high risk of CVD | ≥18 | Not specified |
| Foot exam completed by a nurse | All patients at high risk of CVD | ≥18 | Not specified |
| HbA1c or blood glucose target(s) | All patients at high risk of CVD | ≥18 | Not specified |
| Instructions for home blood glucose monitoring | All patients at high risk of CVD | ≥18 | Not specified |
| Instructions for home blood glucose monitoring received | All patients at high risk of CVD | ≥18 | Not specified |
| Instructions for home BP monitoring | All patients at high risk of CVD | ≥18 | Not specified |
| Instructions for home BP monitoring received | All patients at high risk of CVD | ≥18 | Not specified |
| LDL-C target(s) achieved | All patients at high risk of CVD | ≥18 | Not specified |
| Pharmaceutical opinion on BP medication | All patients at high risk of CVD | ≥18 | Not specified |
| Pharmaceutical opinion on BP medication if the adherence to pharmacological treatment is sub-optimal (<80% or >120%) | All patients at high risk of CVD | ≥18 | Not specified |
| Pharmaceutical opinion on diabetes medication if the adherence to pharmacological treatment is sub-optimal (<80% or >120%) | All patients at high risk of CVD | ≥18 | Not specified |
| Pharmaceutical opinion on diabetes treatment | All patients at high risk of CVD | ≥18 | Not specified |
| Pharmaceutical opinion on lipid lowering treatment | All patients at high risk of CVD | ≥18 | Not specified |
| Pharmaceutical opinion on lipid-lowering medication if the adherence to pharmacological treatment is sub-optimal (<80% or >120%) | All patients at high risk of CVD | ≥18 | Not specified |
| Recommendation for a foot exam by a nurse | All patients at high risk of CVD | ≥18 | Not specified |
| Recommendation for a retina examination | All patients at high risk of CVD | ≥18 | Every 2 years |
| Recommendation for blood glucose diary | All patients at high risk of CVD | ≥18 | Not specified |
| Recommendation for BP diary | All patients at high risk of CVD | ≥18 | Not specified |
| Retina examination | All patients at high risk of CVD | ≥18 | Every 2 years |
| Alcohol consumption target(s) | All patients at moderate or high risk of CVD | ≥18 | Not specified |
| Physical activity target(s) | All patients at moderate or high risk of CVD | ≥18 | Not specified |
| Recommendation for lifestyle changes to increase physical activity | All patients at moderate or high risk of CVD | ≥18 | Not specified |
| Recommendation for lifestyle changes to reduce alcohol consumption | All patients at moderate or high risk of CVD | ≥18 | Not specified |
| Recommendation for lifestyle changes to reduce fat and cholesterol intake | All patients at moderate or high risk of CVD | ≥18 | Not specified |
| Recommendation for lifestyle changes to reduce sodium intake | All patients at moderate or high risk of CVD | ≥18 | Not specified |
| Recommendation for lifestyle changes to reduce stress | All patients at moderate or high risk of CVD | ≥18 | Not specified |
| Recommendation for lifestyle changes to reduce weight and/or waist circumference | All patients at moderate or high risk of CVD | ≥18 | Not specified |
| Recommendations for lifestyle changes to stop smoking | All patients at moderate or high risk of CVD | ≥18 | Not specified |
| Record of alcohol consumption | All patients at moderate or high risk of CVD | ≥18 | Not specified |
| Record of body mass index | All patients at moderate or high risk of CVD | ≥18 | Not specified |
| Record of estimated cardiovascular disease risk using Framingham score | All patients at moderate or high risk of CVD | ≥18 | Not specified |
| Record of foods rich in fat and cholesterol consumption | All patients at moderate or high risk of CVD | ≥18 | Not specified |
| Record of physical activity intensity level | All patients at moderate or high risk of CVD | ≥18 | Not specified |
| Record of salty foods or salt consumption | All patients at moderate or high risk of CVD | ≥18 | Not specified |
| Record of smoking status | All patients at moderate or high risk of CVD | ≥18 | Not specified |
| Record of stress intensity level | All patients at moderate or high risk of CVD | ≥18 | Not specified |
| Record of waist circumference | All patients at moderate or high risk of CVD | ≥18 | Not specified |
| Smoking cessation target(s) | All patients at moderate or high risk of CVD | ≥18 | Not specified |
| Waist circumference target(s) | All patients at moderate or high risk of CVD | ≥18 | Not specified |
| Weight and/or body mass index target(s) | All patients at moderate or high risk of CVD | ≥18 | Not specified |
| Record of prescribing aspirin | All patients | All | Not specified |
| Record of lipid lowering medication | All patients at high risk of CVD | ≥18 | Not specified |
| Record of lipid profile | All patients at high risk of CVD | ≥18 | Not specified |
| Record of referral to dietician or weight loss program | All obese patients | ≥18 | Not specified |
| Record of waist circumference | All obese patients | ≥18 | Not specified |
| Advice for healthy diet | All patients at high risk of CVD | All | Every 12 months |
| Advice for physical activity | All patients at high risk of CVD | All | Every 12 months |
| Advice for smokers to quit smoking | All patients at high risk of CVD | All | Every 12 months |
| Control of BP (mean BP level of maximal 3 measures ≤140/90 mmHg) | All patients at high risk of CVD | All | Not specified |
| Control of total cholesterol level ( ≤5 mmol/l) | All patients at high risk of CVD | All | Not specified |
| Record of blood glucose (random or fasting) | All patients at high risk of CVD | All | Every 12 months |
| Record of BMI or weight | All patients at high risk of CVD | All | Every 12 months |
| Record of BP | All patients at high risk of CVD | All | Every 12 months |
| Record of cholesterol | All patients at high risk of CVD | All | Every 12 months |
| Record of physical activity | All patients at high risk of CVD | All | Every 12 months |
| Record of smoking status | All patients at high risk of CVD | All | Every 12 months |
| Physical body mass index or weight recorded | All patients at high risk of CVD | All | Not specified |
| Record of advice on smoking | All patients at high risk of CVD | All | Not specified |
| Record of blood glucose fasting or random | All patients at high risk of CVD | All | Not specified |
| Record of body mass index ≥30 kg/m2 | All patients at high risk of CVD | All | Not specified |
| Record of BP uncontrolled at >140/90 mmHg | All patients at high risk of CVD | All | Not specified |
| Record of BP | All patients at high risk of CVD | All | Not specified |
| Record of cholesterol | All patients at high risk of CVD | All | Not specified |
| Record of diet advice | All patients at high risk of CVD | All | Not specified |
| Record of motivational status of smokers | All patients at high risk of CVD | All | Not specified |
| Record of physical activity advice | All patients at high risk of CVD | All | Not specified |
| Record of physical activity status | All patients at high risk of CVD | All | Not specified |
| Record of smoker status | All patients at high risk of CVD | All | Not specified |
| Record of smokers being advised to quit | All patients at high risk of CVD | All | Not specified |
| Record of uncontrolled blood glucose fasting >6.1 or random >10.0 mmol/l | All patients at high risk of CVD | All | Not specified |
| Record of uncontrolled cholesterol total ≥5 mmol/l | All patients at high risk of CVD | All | Not specified |
| Record of 2 BP measures | All patients at high risk of CVD | ≥18 | Not specified |
| Record of 2 BP measures | All patients at high risk of CVD | All | Not specified |
| Record of lipid profile | All patients at high risk of CVD | All | Not specified |
| Record of lipid profile | All patients at high risk of CVD | ≥18 | Not specified |
| Record of referral to dietician or weight loss program | All patients at high risk of CVD | ≥18 | Not specified |
| Record of smoking status | All patients at high risk of CVD | ≥18 | Not specified |
| Record of smoking status | All patients at high risk of CVD | All | Not specified |
| Record of waist circumference | All patients at high risk of CVD | All | Not specified |
| Record of waist circumference | All patients at high risk of CVD | ≥18 | Not specified |
| The number of patients whose notes record smoking status in the preceding 12 months. | The number of patients with any or any combination of the following conditions: CHD, PAD, stroke or TIA, hypertension, diabetes, COPD, CKD, asthma, schizophrenia, bipolar affective disorder or other psychoses. | All | Every 12 months |
| The number of patients in the denominator who have had a BMI recorded in the preceding 12 months. | The number of patients with coronary heart disease, stroke or TIA, diabetes, hypertension, peripheral arterial disease, heart failure, COPD, asthma and/ or rheumatoid arthritis. | >18 | Every 12 months |
| The number of patients in the denominator whose notes record smoking status in the preceding 12 months. | The number of patients with any or any combination of the following conditions: CHD, PAD, stroke or TIA, hypertension, diabetes, COPD, CKD or asthma. | All | Every 12 months |
| The number of patients who have a record of an offer of support and treatment for smoking within the preceding 12 months | The number of patients with any or any combination of the following conditions: CHD, PAD, stroke or TIA, hypertension, diabetes, COPD, CKD, asthma who are recorded as current smokers. | All | Every 12 months |
| The number of patients who have a record of an offer of support and treatment for smoking within the preceding 12 months. | The number of patients with any or any combination of the following conditions: CHD, PAD, stroke or TIA, hypertension, diabetes, COPD, CKD, asthma, schizophrenia, bipolar affective disorder or other psychoses who are recorded as current smokers. | All | Every 12 months |
| The number of patients who were prescribed oral anticoagulants in the 6 months leading up to and including the payment period end date. | The number of patients with most recent CHA2DS2-VASc stroke risk assessment score of 2 or more and those who did not have a CHA2DS2-VASc but they had a CHADS2 stroke risk assessment score of 2 or more. | All | Not specified |
| ≥1 advice about a low-risk alcohol drinking pattern (men ≤ 2 units per day; women ≤ 1 unit per day) offered, unless a no or low risk alcohol drinking | All patients at high risk of CVD | All | Every 12 months |
| ≥1 offered specific advice about lifestyle | All patients at CVD event risk of ≥ 20 % (>10 years) | All | Every 12 months |
| ≥1 risk re-assessed | All patients at high risk of CVD | ≥18 | Every 5 years |
| Record of offered ≥1 diet advice | All patients at high risk of CVD | All | Every 12 months |
| Record of serum cholesterol controlled to < 5.0mmol/l (approx. 195mg/dl) | All patients at high risk of CVD | ≥18 | Not specified |
| Statin unless there are no other risk factors who have sustained proteinuria | All patients at high risk of CVD | ≥18 | Not specified |
| ≥1 offered advice about regular physical activity unless contraindicated | All patients at moderate risk of CVD | All | Every 12 months |
| CVD risk re-assessed | All patients with a CVD event risk score: < 10% (>10 years) | All | Every 5-10 years |
| CVD risk re-assessed | All patients with a CVD event risk score: 10%-15% (>10 years) | All | Every 5 years |
| Record of offered drug therapy for persistent BP elevation of >160 /100 mmHg | All hypertensive patients | All | Not specified |
| Record of smoking status | All patients at risk of RHD or ARF | All | Not specified |
| Health-related Quality of life—EQ5D (version 5L with Australian standardized weights) | All patients with moderate to high risk of CVD | >18 | Every 12 months |
| Meeting individual targets for BP and LDL cholesterol | All patients with moderate to high risk of CVD | >18 | Every 12 months |
| Obesity— body mass index >30 kg/m2 | All patients with moderate to high risk of CVD | >18 | Every 12 months |
| Patients whose BP AND fasting low-density lipoprotein (LDL) cholesterol were meeting Australian guideline targets (defined as: ≤130/80 mmHg for CVD, diabetes, or albuminuria or ≤140/90 mmHg for all others, AND LDL-cholesterol <2.0 mmol/L) | All patients with moderate to high risk of CVD | >18 | Every 12 months |
| Record of fruit and vegetable intake, fish, salt, and saturated fat intake—self-reported portions consumed in 7 days prior | All patients with moderate to high risk of CVD | >18 | Every 12 months |
| Self-reported physical activity based on World Health Organization (WHO) Global Physical Activity Questionnaire | All patients with moderate to high risk of CVD | >18 | Every 12 months |
| Recording of 1 BP reading | All patients at high risk of CVD | >40 | Not specified |
| Recording of 2 BP readings | All patients at high risk of CVD | >40 | Not specified |
| Recording of albumin to creatinine ratio | All patients at high risk of CVD | >40 | Not specified |
| Recording of eGFR | All patients at high risk of CVD | >40 | Not specified |
| Recording of fasting blood glucose | All patients at high risk of CVD | >40 | Not specified |
| Recording of lipid profile | All patients at high risk of CVD | >40 | Not specified |
| Recording of smoking status checked | All patients at high risk of CVD | >40 | Not specified |
| Recording of waist circumference | All patients at high risk of CVD | >40 | Not specified |
| Recording of HbA1c values <7.0% | All patients at high risk of CVD | 18-80 | Not specified |
| Recording of postprandial plasma glucose < 7.5 mmol/L | All patients at high risk of CVD | 18-80 | Not specified |
| Recording of BP < 130/80 mmHg | All patients at high risk of CVD | 18-80 | Not specified |
| Recording of BP <140/90 mmHg | All patients at high risk of CVD | 18-80 | Not specified |
| Recording of fasting plasma glucose < 6 mmol/L | All patients at high risk of CVD | 18-80 | Not specified |
| Recording of total cholesterol < 3.5 | All patients at high risk of CVD | 18-80 | Not specified |
| Recording of total cholesterol <5.0 mmol/L | All patients at high risk of CVD | 18-80 | Not specified |
| Current smokers who have a record of an offer of support and treatment within the preceding 12 months | Patients with any or any combination of the following conditions: CHD, PAD, stroke or TIA, hypertension, diabetes, COPD, CKD, asthma, schizophrenia, bipolar affective disorder or other psychoses | All | Every 12 months |
| Record of smoking status | Patients with any or any combination of the following conditions: CHD, PAD, stroke or TIA, hypertension, diabetes, COPD, CKD, asthma, schizophrenia, bipolar affective disorder or other psychoses | > 75 | Every 12 months |
| Recording of patients who were prescribed/self-report taking statins | All patients at high risk of CVD | ≥18 | Not specified |
| Prescription of lipid-lowering medications to 10-y CVD risk of ≥20% Framingham CVD risk score | All patients at risk of CVD | ≥18 | Not specified |
| Achieved either BP or LDL cholesterol targets, or for whom treatment was intensified (newly prescribed or additional antiplatelet, BP-lowering, or lipid-lowering drugs) | All untreated patients | ≥18 | Not specified |
| Mean changes in systolic and diastolic BP and lipid levels for undertreated patients at high CVD risk | All untreated patients | ≥18 | Not specified |
| Received a new prescription or intensified prescribing of BP- or lipid-lowering or antiplatelet medications | All patients | ≥18 | Not specified |
| Record of achieved both BP and LDL cholesterol targets | All patients with high risk of CVD | ≥18 | Not specified |
| Undertreated patients with high CVD  risk who achieved both BP≤ 140/90 mmHg; ≤ 130/80 mmHg in people with diabetes or albuminuria and LDL cholesterol goals (< 2.0 mmol/L) by study end. | All untreated patients | ≥18 | Not specified |
| Prescribing a traditional oral NSAID | All patients without co-prescription of gastro-protection | ≥75 | Not specified |
| Prescribing an oral NSAID in patients prescribed both a diuretic and an angiotensin-converting-enzyme inhibitor (ACE inhibitor) or angiotensin receptor blocker (ARB) | All patients prescribed both a diuretic and an angiotensin-converting-enzyme inhibitor (ACE inhibitor) or angiotensin receptor blocker (ARB) | All | Not specified |
| Prescribing of a traditional oral NSAID and aspirin | All patients without co-prescription of gastro-protection | ≥65 | Not specified |
| Prescribing of aspirin and clopidogrel | All patients without co-prescription of gastro-protection | ≥65 | Not specified |
| Prescribing of warfarin and a traditional oral NSAID without co-prescription of gastro-protection | All patients at risk of CVD | All | Not specified |
| Prescribing of warfarin and low-dose aspirin or clopidogrel without co-prescription of gastro-protection | All patients at risk of CVD | All | Not specified |
| **Diabetes (n=130)** | | | |
| Record of HbA1c checked | All diabetic patients | ≥18 | Every 12 months |
| Patients with type 2 diabetes and chronic kidney disease who are prescribed SGLT2 inhibitors | All patients with type 2 diabetes and chronic kidney disease | ≥18 | Not specified |
| Record of patients who have no contraindication, refusal, or history of intolerance to renin–angiotensin– aldosterone system inhibitors | All patients with diabetes and chronic kidney disease or hypertension | ≥18 | Not specified |
| Available written protocols to encourage and facilitate disease self-measurement for patients with hypertension and/or diabetes | All healthcare centres or units | NA | Not specified |
| Meet the established goals for optimum management: (a) Waist circumference; (b) BP; (c) Total cholesterol (d) Microalbumin | All patients with T2D | All | Not specified |
| Record of (a) serum lipids and (b) microalbumin checked | All patients with T2D | All | Every 12 months |
| Record of HbA1c checked | All patients with T2D | All | Every 6 months |
| Record of HbA1c of <7% | All patients with T2D | All | Not specified |
| Written annual cycle of care plan in their records | All patients with T2D | All | Every 12 months |
| Record of BMI | All diabetic patients | All | Every 12 months |
| Record of diagnosis of proteinuria or micro-albuminuria who are treated with ACE inhibitors (or A2 antagonists) +I146 | All diabetic patients | All | Not specified |
| Record of HbA1c is ≤8 | All diabetic patients | All | Every 12 months |
| Record of influenza immunisation | All diabetic patients | All | Not specified |
| Record of last BP is ≤145/85 mmHg | All diabetic patients | All | Every 12 months |
| Record of last measured total cholesterol is ≤5 mmol/l | All diabetic patients | All | Every 12 months |
| Record of neuropathy testing | All diabetic patients | All | Every 12 months |
| Record of the presence or absence of peripheral pulses | All diabetic patients | All | Every 12 months |
| Record of blood pressure measurement | All diabetic patients | All | Every 6 months |
| The number of regular clients who are recorded as having type 1 or type 2 diabetes AND who are immunised against influenza | The total number of regular clients who are recorded as having type 1 or type 2 diabetes | All | Every 12 months |
| Record BMI | All diabetic patients | ≥18 | Not specified |
| Record of blood glucose level | All diabetic patients | ≥18 | Not specified |
| Record of serum creatinine testing | All diabetic patients | ≥18 | Not specified |
| Record of smoking cessation advice or referral to a specialist | All diabetic patients | All | Every 12 months |
| Record of smoking status | All diabetic patients | All | Every 12 months |
| Achievement of cholesterol targets of a total cholesterol ≤5 mmol/l | All diabetic patients | All | Every 12 months |
| Achieved target level of LDL-C <70 mg/dl | All diabetic patients | All | Not specified |
| Change in lipid-lowering therapy at the time of consultation when LDL-C above the target level was determined | All diabetic patients | All | Not specified |
| Record of last HbA1C level | All diabetic patients | All | Not specified |
| Record of last LDL-C level | All diabetic patients | All | Not specified |
| Scheduling of a reassessment consultation and monitoring of lipid parameters if target LDL-C level was not achieved | All diabetic patients | All | Every 3 months |
| Inhibitors of the renin-angiotensin-aldosterone system (RAAS; ACE-I or ARB or Renin-Inhibitors [RI]), calcium channel blockers, β-blockers or thiazides or a combination of these drugs for Arterial hypertension with BP above target (systolic BP >140 mmHg in multiple or 24 h-measurements or >160 mmHg in single measurement) | All diabetic patients | All | Not specified |
| Record of Metformin for Diabetes mellitus type 2 (HbA1c >53 mmol/mol (7%)) | All diabetic patients | All | Not specified |
| The percentage of patients with any of any combination of the following condition: CHD, stroke or TIA, hypertension, diabetes, COPD or asthma, who smoke and whose notes contain a record that smoking cessation advice or referral to a specialist service, where available, has been offered within the previous 15 months | All patients with any of these condition : CHD, stroke or TIA, hypertension, diabetes, COPD or asthma, | >65; male | Every 12 months |
| Record of last HbA1C is ≤7.5 (or equivalent test/reference range depending on laboratory) | All diabetic patients | >65; male | Every 12 months |
| Record of last BP reading is ≤145/85 mm Hg | All diabetic patients | >65; male | Not specified |
| Record of measured total cholesterol level is ≤5 mmol/L | All diabetic patients | >65; male | Every 12 months |
| Number of persons with the PCG “diabetes mellitus” and with ≥1 control of kidney per year | Number of persons with the PCG “diabetes mellitus” | ≥18 | Every 12 months |
| Number of patients in participating practices with diabetes with a glycated haemoglobin ≤7 mmol/l | All diabetic patients | All | Not specified |
| Number of patients with measured total cholesterol <4 mmol/litre | All diabetic patients | All | Not specified |
| Number of patients with recorded BP ≤130/80 | All diabetic patients | All | Not specified |
| Patients with diabetes service incentive payment claimed | All diabetic patients | All | Not specified |
| Record of BP ≤145/85 mm Hg | All diabetic patients | All | Not specified |
| Record of HbA1c ≤7.5 | All diabetic patients | All | Not specified |
| Record of last total cholesterol was <5 mmol/l | All diabetic patients | All | Not specified |
| Record of 2 BP measures | All diabetic patients | ≥18 | Not specified |
| Record of 2 haemoglobin A1c tests | All diabetic patients | ≥18 | Not specified |
| Record of albumin-to-creatinine ratio (ACR) | All diabetic patients | ≥18 | Not specified |
| Record of estimated glomerular filtration rate (eGFR) | All diabetic patients | ≥18 | Not specified |
| Record of glycaemic control medication | All diabetic patients | ≥18 | Not specified |
| With a fasting or direct low-density lipoprotein cholesterol level ≥190 mg/dL | All diabetic patients | ≥18 | Not specified |
| With a fasting or direct low-density lipoprotein cholesterol level 70-189 mg/dL, who were prescribed or are already on statin medication therapy | All diabetic patients | ≥18 | Every 12 months |
| Use a system for recalling patients with diabetes | All practices | NA | Not specified |
| Record of glycaemic control medication | All diabetic patients | ≥18 | Not specified |
| Record of recommended 2 HbA1c tests | All diabetic patients | ≥18 | Not specified |
| Patients treated with statins (unless there is a contraindication). | Patients with a new diagnosis of hypertension or type 2 diabetes | 25-84 | Every 12 months |
| Patients who have had consultation for a full formal cardiovascular disease risk assessment between 3 months before or 3 months after date of diagnosis | Patients with a new diagnosis of hypertension or type 2 diabetes | 25-84 | Every 12 months |
| Record of achieved BP target of <130/80 mmHg | All diabetic patients | ≥18 | Not specified |
| ≥1 offered advice about regular physical activity unless contraindicated | All diabetic patients | All | Every 12 months |
| ≥1 risk assessed at diagnosis and re-assessed | All diabetic patients | All | Every 5 years |
| Record of ≥1 BP measurement | All diabetic patients | All | Every 12 months |
| Record of ≥1 weight or BMI | All diabetic patients | All | Every 12 months |
| Record of offered ≥1 diet advice | All diabetic patients | All | Every 12 months |
| Record of serum cholesterol controlled as per standard (<5 mmol/l or approx. 195 mg/dl) | All diabetic patients | ≥40 | Not specified |
| Diagnosis of diabetes should be clearly identifiable from the paper and/or electronic record | All practices | NA | Not applicable |
| Screening for retinopathy in patients with diabetes | Active patients with diabetes seen in the past 2 years | All | 2 years |
| Record of achieved target of HbA1c ≤ 7.0% | All diabetic patients | ≥16 | Every 12 months |
| Record of achieved target of BP ≤130/80 mmHg | All diabetic patients | ≥16 | Every 12 months |
| Record of achieved target of cholesterol ≤ 4 mmol | All diabetic patients | ≥16 | Every 12 months |
| Record of ACR level | All diabetic patients | ≥16 | Every 12 months |
| Record of BMI status | All diabetic patients | ≥16 | Every 12 months |
| Record of ischaemic heart disease status | All diabetic patients | ≥16 | Every 12 months |
| Record of prescribed medication for diabetes | All diabetic patients | ≥16 | Every 12 months |
| Record of prescription of antidepressant drugs | All diabetic patients | ≥16 | Not specified |
| Record of recent albumin/creatinine ratio (ACR) | All diabetic patients | ≥16 | Every 12 months |
| Record of recent BP | All diabetic patients | ≥16 | Every 12 months |
| Record of recent Haemoglobin A1c (HbA1c) | All diabetic patients | ≥16 | Every 12 months |
| Record of recent total cholesterol | All diabetic patients | ≥16 | Every 12 months |
| Record of renal disease status | All diabetic patients | ≥16 | Every 12 months |
| Record of screened for depression or other mental illness using standard screening tools such as K-5, K-6, K-10, PHQ-2+, PHQ-9 and Edinburgh Postnatal Depression Screening tool (EPDS) | All diabetic patients | ≥16 | Every 12 months |
| Record of smoking status | All diabetic patients | ≥16 | Every 12 months |
| Cholesterol measurement | All bereavement patients with diabetes | ≥60 | Every 12 months |
| Depression assessment | All bereavement patients with diabetes | ≥60 | Every 12 months |
| Lipid-lowering medication prescription | All bereavement patients with diabetes | ≥60 | Every 12 months |
| Record of HbA1c measurement | All bereavement patients with diabetes | ≥18 | Every 12 months |
| Renin–angiotensin system drug prescription | All bereavement patients with diabetes | ≥60 | Every 12 months |
| Blood pressure measurement | All bereavement patients with diabetes or hypertension | ≥60 | Every 12 months |
| Electrolyte measurement (Must have prescription for diuretics or angiotensin-converting enzyme inhibitor in the year before and year after bereavement) | All bereavement patients with diabetes or hypertension | ≥60 | Every 12 months |
| Influenza vaccination | All bereavement patients with diabetes or hypertension | ≥60 | Every 12 months |
| Record of ≥1 encounters with prescription of statin therapy to patients with diagnosis of diabetes, LDL 70–189 mg/dL, without diagnosis of ASCVD | All diabetic patients | ≥18 | Every 12 months |
| Recording of 2 BP reading | All diabetic patients | ≥18 | Not specified |
| Recording of albumin to creatinine ratio | All diabetic patients | ≥18 | Not specified |
| Recording of eGFR | All diabetic patients | ≥18 | Not specified |
| Recording of fasting blood glucose | All diabetic patients | ≥18 | Not specified |
| Recording of HbA1c | All diabetic patients | ≥18 | Not specified |
| Recording of lipid profile | All diabetic patients | ≥18 | Not specified |
| Recording of prescribed glycaemic medication | All diabetic patients | ≥18 | Not specified |
| Recording of BP < 130/80 mmHg | All diabetic patients | ≥18 | Not specified |
| Recording of total cholesterol < 4.5 mmol/L | All diabetic patients | ≥18 | Not specified |
| Controlled hypertension of <140/80 mmHg | All diabetic patients | ≥30 | Not specified |
| Record of fundoscopy | All T2DM patients | ≥30 | Every 12 months |
| Prescription of alpha-blocker as a single agent | All hypertensive patients with diabetes | ≥65 | Not specified |
| Prescription of beta-blocker as a single agent | All hypertensive patients with T2DM or dyslipidaemia | ≥30 | Not specified |
| Prescription of diuretic as a single agent | All hypertensive patients with T2DM or dyslipidaemia | ≥30 | Not specified |
| Prescription of angiotensin converting enzyme inhibitor (ACEI) or angiotensin receptor blocker (ARB) | All T2DM patients with proteinuria | ≥30 | Not specified |
| Maintains a register of all patients aged 17 or over with diabetes mellitus, which specifies the type of diabetes where a diagnosis has been confirmed | All practices | >17 | Not specified |
| Record of a foot examination and risk classification: 1) low risk (normal sensation, palpable pulses), 2) increased risk (neuropathy or absent pulses), 3) high risk (neuropathy or absent pulses plus deformity or skin changes in previous ulcer) or 4) ulcerated foot within the preceding 12 months | Diabetic patients | < 75 | Every 12 months |
| Record of being referred to a structured education programme within 9 months after entry on to the diabetes register | Diabetic patients | < 75 | Every 9 months |
| Record of diagnosis of nephropathy (clinical proteinuria) or microalbuminuria who are currently treated with an ACE-I (or ARBs) | Diabetic patients | < 75 | Not specified |
| Record of last blood pressure reading (measured in the preceding 12 months) is 140/80 mmHg or less | Diabetic patients | < 75 | Every 12 months |
| Record of last blood pressure reading (measured in the preceding 12 months) is 150/90 mmHg or less | Diabetic patients | < 75 | Every 12 months |
| Record of last IFCC-HbA1c is 59 mmol/mol or less in the preceding 12 months | Diabetic patients | < 75 | Every 12 months |
| Record of last IFCC-HbA1c is 64 mmol/mol or less in the preceding 12 months | Diabetic patients | < 75 | Every 12 months |
| Record of last IFCC-HbA1c is 75 mmol/mol or less in the preceding 12 months | Diabetic patients | < 75 | Every 12 months |
| Record of last measured total cholesterol (measured within the preceding 12 months) is 5 mmol/l or less | Diabetic patients | < 75 | Every 12 months |
| Record of patients who have had influenza immunisation | Diabetic patients | < 75 | Not specified |
| Record of achieved treatment target by patients on glucose lowering therapy | All patients on glucose lowering therapy | ≥18 | Not specified |
| Record of achieved treatment target of HbA1c ≤ 7.5% | All diabetic patients without CVD | ≥18 | Not specified |
| Record of anti-hypertensive therapy if their BP > 140/90 mmHg | All diabetic patients without CVD | ≥18 | Not specified |
| Record of cholesterol/HDL-cholesterol ratio < 4.0 | All diabetic patients | ≥18 | Not specified |
| Record of lipid lowering therapy if total cholesterol/HDL-cholesterol ratio > 5.0 and they had ≥1 additional risk factor (current antihypertensive therapy, smoking, microalbuminuria or a family history of premature CVD) | All diabetic patients without CVD | ≥18 | Not specified |
| Recording of patients whose most recent HbA1c measurement was <7% | All primary healthcare patients with diabetes (Type I or Type II) | ≥18 | Every 12 months |
| Recording of patients who are taking angiotensin-converting enzyme (ACE)-inhibitors or angiotensin II receptor blockers (ARBs) | All primary healthcare patients with diabetes (Type I or Type II) | ≥18 | Not specified |
| Recording of patients who are taking anti-diabetic medications (oral agents or insulin | All primary healthcare patients with diabetes (type I or type II) | ≥18 | Not specified |
| Recording of patients who were prescribed statins | All primary healthcare patients with diabetes (Type I or Type II) | ≥18 | Not specified |
| Record of last BP is ≤145/85 | All diabetic patients | ≥18 | Every 12 months |
| Record of last HbA1c is ≤10 (or equivalent test/reference range depending on local laboratory) | All diabetic patients | ≥18 | Every 12 months |
| Record of last HbA1c is ≤7.4 (or equivalent test/reference range depending on local laboratory) | All diabetic patients | ≥18 | Every 12 months |
| Record of last measured total cholesterol is ≤5mmol/l | All diabetic patients | ≥18 | Every 12 months |
| Achieved recommended target levels for HbA1c ≤59 mmol/mol and cholesterol ≤5 mmol/l and BP<140/80 mmHg (or <130/80 mmHg if kidney, eye or cerebrovascular damage) | All diabetic patients | All | Not specified |
| Composite indicator Record of the BP measurement, HbA1c, lipids, kidney function (urine albumin & protein), foot review, retinopathy, BMI, and current smoking | All diabetic patients | All | 6-15 months |
| **Hypertension (n=85)** | | | |
| Record of patients who have no contraindication, refusal, or history of intolerance to renin–angiotensin– aldosterone system inhibitors | All patients with diabetes and chronic kidney disease or hypertension | ≥18 | Not specified |
| Available written protocols to encourage and facilitate disease self-measurement for patients with hypertension and/or diabetes | All healthcare centres or units | NA | Not specified |
| Achieved BP level of <140/90 mm/Hg | All hypertensive patients | ≥18 | Not specified |
| Patients were assumed to be non-diabetic if diabetes status was not specified | All hypertensive patients | All | Not specified |
| Patients were assumed to be non-smokers if smoking status was not specified | All hypertensive patients | All | Not specified |
| Proportions of patients with BP lowering drug prescribed | All hypertensive patients | ≥18 | Not specified |
| Record of fasting glucose tested | All hypertensive patients | All | Not specified |
| Record of medical diagnosis of CVD | All hypertensive patients | All | Not specified |
| Record of most recent SBP/DBP | All hypertensive patients | All | Not specified |
| Record of recent BP measurement was at normal range (< 140/90 mmHg) | All hypertensive patients | All | Not specified |
| Record of smoking status recorded | All hypertensive patients | All | Not specified |
| Record of total cholesterol tested | All hypertensive patients | All | Not specified |
| Record of two BP measurements | All hypertensive patients | All | Every 12 months |
| Recorded all risk factors (age, sex, BP, smoking status, diabetes status, and total blood cholesterol) to calculate the WHO/ISH CVD risk score | All hypertensive patients | ≥40 | Every 2 years |
| Recorded calculated WHO/ISH ≥ 30 % or/and a history of CVD | All hypertensive patients | ≥40 | Not specified |
| Recorded risk score based on age, sex, BP, smoking status, diabetes status, and total blood cholesterol | All hypertensive patients | ≥40 | Not specified |
| Record of changes in systolic BP after each therapeutic change | All hypertensive patients | 18-85 | Every 6 and 12 months |
| Record of antihypertensive medications | All hypertensive patients | 18-85 | Every 6 and 12 months |
| Record of baseline height and weight for body mass index (kg/m2) | All hypertensive patients | 18-85 | Every 6 months |
| Record of controlled BP (<140/90 mm Hg) | All hypertensive patients | 18-85 | Every 6 and 12 months |
| Record of therapeutic inertia index (calculated as the proportion of visits in which no changes were made to the number or dose of antihypertensive medications relative to the number of visits with uncontrolled BP) | All hypertensive patients | 18-85 | Every 6 and 12 months |
| Record of smoking cessation advice or referral to a specialist service, where available, has been offered within the previous 15 months | All patients with any of these conditions: CHD, stroke or TIA, hypertension, diabetes, COPD or asthma, | >65; male | Every 15 months |
| Record of last BP reading is ≤150/90 mm Hg | All hypertensive patients | ≥18 | Every 9 months |
| Record of smoking status | All hypertensive patients | ≥18 | Not specified |
| Record of 2 BP measures | All hypertensive patients | ≥18 | Not specified |
| Record of anti-hypertensive medication | All hypertensive patients | ≥18 | Not specified |
| Record of controlled BP (<140/90 mm Hg) | All hypertensive patients | 18-85 | Not specified |
| Use a system for recalling patients with hypertension | All practices | ≥18 | Not specified |
| Patients treated with statins (unless there is a contraindication). | The number of patients aged between 25 and 84 years with a new diagnosis of hypertension or type 2 diabetes, recorded in the preceding 12 months | 25-84 | Every 12 months |
| Number of patients who have had consultation for a full formal cardiovascular disease risk assessment between 3 months before or 3 months after date of diagnosis | The number of patients aged between 25 and 84 years with a new diagnosis of hypertension or type 2 diabetes, recorded in the preceding 12 months (excluding those with pre-existing cardiovascular disease, chronic kidney disease, familial hypercholesterolaemia or type 1 diabetes). | 25-84 | Every 12 months |
| The number of patients who have a record of a 12-lead ECG performed in the three months before or after the date of entry to the hypertension register | The number of patients with a new diagnosis of hypertension | All | Within 3 months |
| The number of patients who have a record of a brief intervention for increasing physical activity in the preceding 15 months. | The number of patients on the hypertension register aged between 16 and 74 years who scored ‘less than active’ on the GPPAQ assessment tool in the preceding 15 months. | 16-74 | Every 12 months |
| The number of patients who have a record of a test for haematuria in the three months before or after the date of entry to the hypertension register. | The number of patients with a new diagnosis of hypertension. | All | Within 3 months |
| The number of patients who have a record of an assessment of physical activity using the GPPAQ assessment tool in the preceding 15 months. | The number of patients on the hypertension register aged between 16 and 74 years. | 16-74 | Every 12 months |
| The number of patients who have a record of urinary albumin: creatinine ratio test in the three months before or after the date of entry to the hypertension register. | The number of patients with a new diagnosis of hypertension | >18 | Within 3 months |
| The number of patients whose diagnosis has been confirmed by ambulatory blood pressure monitoring (ABPM) or home blood pressure monitoring (HBPM) in the three months before entering on to the register. | The number of patients with a new diagnosis of hypertension | All | Within 3 months |
| The number of patients who are currently treated with renin-angiotensin system antagonists. | The number of patients on the CKD register with hypertension and proteinuria | All | Not specified |
| Record of achieved BP target of <140/90mmHg | All patients | ≥45 | Not specified |
| Record of BMI status | All patients | ≥45 | Not specified |
| Record of excessive intake of alcohol | All patients | ≥45 | Not specified |
| Record of physical activity | All patients | ≥45 | Not specified |
| Record of smoking status | All patients | ≥45 | Not specified |
| Record of treatment compliance of hypertensive medications | All hypertensive patients | ≥45 | Not specified |
| Record of use of antihypertensive treatment | All hypertensive patients | ≥45 | Not specified |
| Record of use of associations of hypertensive drugs of the same class, with the no exception of loop or thiazide diuretics with spironolactone. | All hypertensive patients | ≥45 | Not specified |
| Record of use of monotherapy medications such as angiotensin-converting enzyme inhibitors (ACEi); angiotensin receptor blockers (ARB); adrenergic beta-blockers; thiazide, loop, and potassium-sparing diuretics; or calcium channel blockers. | All hypertensive patients | ≥45 | Not specified |
| Record of use of triple therapy, prescribed one diuretic | All hypertensive patients | ≥45 | Not specified |
| Offered drug therapies to unless contraindicated or intolerant | All patients with sustained (>3 times) SBP ≥ 160 mmHg or sustained DBP ≥ 100 mmHg despite up to six months of non-pharmacological measures | ≥18 | Not specified |
| Record of offered ≥1 diet advice | All hypertensive patients | All | Every 12 months |
| Diagnosis of hypertension should be clearly identifiable from the paper and/or electronic record | All practices | NA | Not applicable |
| Number of hypertensive patients screened using both eGFR and urinary albumin in the past 12 months. | Number of hypertensive patients seen in the past 12 months. | All | Every 12 months |
| Blood pressure measurement | All bereavement patients with diabetes or hypertension | ≥60 | Every 12 months |
| Electrolyte measurement (Must have prescription for diuretics or angiotensin-converting enzyme inhibitor in the year before and year after bereavement) | All bereavement patients with diabetes or hypertension | ≥60 | Every 12 months |
| Influenza vaccination | All bereavement patients with diabetes or hypertension | ≥60 | Every 12 months |
| Record of ≥1 encounters of BP and was adequately controlled (<140/90) | All hypertensive patients | 18-85 | Every 12 months |
| Recording of 2 BP readings | All hypertensive patients | >40 | Not specified |
| BMI <27.5 kgm^2^ considered as obese for Asians | All hypertensive patients | ≥30 | Not specified |
| Controlled hypertension of <140/90 mmHg | All hypertensive patients | ≥30 | Not specified |
| Controlled hypertension of <150/90 mmHg | All hypertensive patients | ≥80 | Not specified |
| Counselling on diet education, exercise/physical activity, smoking cessation, salt intake, and alcohol intake. | All hypertensive patients | ≥30 | Every 12 months |
| Framingham risk score performed | All hypertensive patients | ≥30 | Every 12 months |
| Record of creatinine level | All hypertensive patients | ≥30 | Every 12 months |
| Record of ECG | All hypertensive patients | ≥30 | Every 12 months |
| Record of fasting lipid profile including total cholesterol, triglycerides, HDL and HDL cholesterol level | All hypertensive patients | ≥30 | Every 12 months |
| Record of fasting/random blood glucose level | All hypertensive patients | ≥30 | Every 12 months |
| Record of fundoscopy | All hypertensive patients | ≥30 | Every 12 months |
| Record of prescribed antihypertensive agents such as calcium channel blocker, angiotensin converting enzyme inhibitor, beta blocker, diuretics, angiotensin receptor blocker, & alpha blocker | All hypertensive patients | ≥18 | Not specified |
| Record of urine albumin level | All hypertensive patients | ≥30 | Every 12 months |
| Record of weight, height, and waist circumference | All hypertensive patients | ≥30 | Every 12 months |
| Recording of SBP & DBP | All hypertensive patients | ≥30 | Every 12 months |
| Prescription of alpha-blocker as a single agent | All hypertensive patients with diabetes | ≥65 | Not specified |
| Prescription of beta-blocker as a single agent | All hypertensive patients with T2DM or dyslipidaemia | ≥30 | Not specified |
| Prescription of diuretic as a single agent | All hypertensive patients with T2DM or dyslipidaemia | ≥30 | Not specified |
| Record of CVD risk assessment score (using an assessment tool agreed with the NHS CB) of ≥20% in the preceding 12 months: the percentage who are currently treated with statins | Patients with a new diagnosis of hypertension aged 30 or over and who have not attained the age of 75, recorded  between the preceding 1 April to 31 March (excluding those with pre-existing CHD, diabetes, stroke and/or TIA), | 30-75 | Every 12 months |
| Record of last blood pressure reading (measured in the preceding 12 months) is 150/90 mmHg or less | All hypertensive patients | < 75 | Every 12 months |
| The contractor establishes and maintains a register of patients with established hypertension | All practices | < 75 | Not specified |
| Achieved treatment target of SBP ≤ 140 mmHg, DBP ≤ 85 mmHg by patients on anti-hypertensive therapy | All hypertensive patients | ≥20 | Not specified |
| Recording of mean number of antihypertensive medications taken | All primary healthcare patients with hypertension taking ≥1 antihypertensive medication | ≥20 | Not specified |
| Recording of patients visited emergency department for hypertension | All primary healthcare patients with hypertension | ≥20 | Not specified |
| Recording of patients who had a BP measure <140mmHg systolic and <90mmHg diastolic | All primary healthcare patients with hypertension | ≥20 | Not specified |
| Recording of prescribed/self-report taking ≥1 anti-hypertension medication | All primary healthcare patients with hypertension | ≥20 | Not specified |
| Prescription of antihypertensive medications to patients with BP ≥160/100 mm Hg | All hypertensive patients | ≥18 | Every 3 years |
| Record of last BP is ≤150/90 | All hypertensive patients | ≥18 | Every 9 months |
| Achieved recommended target levels for BP<150/90 mmHg | All hypertensive patients | ≥18 | Not specified |
| Recording of thyroid stimulating hormone | All hypertensive with non-SMI patients | ≥16 | Not specified |
| Normal BP (150/90 mm Hg) in last 9 months | All hypertensive with SMI patients | ≥16 | Every 9 months |
| Record of last BP | All hypertensive with SMI patients | ≥16 | Every 9 months |
| Recording of Alcohol | All hypertensive with SMI patients | ≥16 | Not specified |
| Recording of BMI | All hypertensive with SMI patients | ≥16 | Not specified |
| Recording of BP | All hypertensive with SMI patients | ≥16 | Not specified |
| Recording of CHD comorbidity | All hypertensive with SMI patients | ≥16 | Not specified |
| Recording of cholesterol | All hypertensive with SMI patients | ≥16 | Not specified |
| Recording of CVD risk factor assessment | All hypertensive with SMI patients | ≥16 | Not specified |
| Recording of diabetes mellitus comorbidity | All hypertensive with SMI patients | ≥16 | Not specified |
| Recording of eGFR | All hypertensive with SMI patients | ≥16 | Not specified |
| Recording of HbA1c | All hypertensive with SMI patients | ≥16 | Not specified |
| Recording of smoking status | All hypertensive with SMI patients | ≥16 | Not specified |
| Recording of thyroid stimulating hormone | All hypertensive with SMI patients | ≥16 | Not specified |
|  |  |  |  |
| **Atrial fibrillation (n=63)** | | | |
| New atrial fibrillation diagnosis | All patients with atrial fibrillation | All | Not specified |
| Patients diagnosed with AF who have a CHA2DS2-VASc score ≥1 for males or ≥2 for females prescribed anticoagulation drug therapy | All patients with AF | All | Not specified |
| Patients newly diagnosed with atrial fibrillation | All patients | All | Not specified |
| Patients prescribed antiplatelet monotherapy | All patients with atrial fibrillation | All | Not specified |
| Patients who have received HAS-BLED risk assessment | All patients | All | Not specified |
| Patients who received CHA2DS2-VASc risk assessment | All patients | All | Not specified |
| Patients with a new or pre-existing diagnosis of atrial fibrillation | All patients | All | Not specified |
| Clinician reported symptom status assessed using a validated tool (e.g. European Heart Rhythm Association symptom score) during the measurement duration. | Number of patients with AF | ≥18 | Not specified |
| Number of documented AF-related thrombo-embolic events during the measurement duration. | Number of patients with AF | ≥18 | Not specified |
| Prescribed one or more antiarrhythmic drugs for rhythm control. | Number of patients with permanent AF | ≥18 | Not specified |
| Record of CHA2DS2-VASc score of ≥1 for men and ≥2 for women and are prescribed anticoagulation for AF | Number of patients with AF who have CHA2DS2-VASc score of ≥1 for men and ≥2 for women | ≥18 | Not specified |
| Record of cognitive function assessed using a validated instrument. | Number of patients with AF | ≥18 | Every 12 months |
| Record of an ECG confirming AF diagnosis. | Number of patients with AF | ≥18 | Not specified |
| Record of bleeding risk assessment documented using a validated bleeding risk score | Number of patients with AF | ≥18 | Every visit |
| Record of CHA2DS2-VASc score documented at the time of diagnosis and at every follow-up appointment. | Number of patients with AF | ≥18 | Every visit |
| Record of CHA2DS2-VASc score of 0 for men and 1 for women and are inappropriately prescribed long-term anticoagulation for AF | Number of patients with AF who have CHA2DS2-VASc score of 0 for men and 1 for women and do not have other indication for anticoagulation | ≥18 | Not specified |
| Record of complete electrical isolation (entrance and exit block) of the pulmonary veins (PVs) during AF catheter ablation procedures | Number of patients with AF treated with catheter ablation procedures | ≥18 | Not specified |
| Record of died due to an invasive procedure for AF management during the measurement duration | Number of patients with AF treated with invasive procedures | ≥18 | Not specified |
| Record of died during the measurement duration | Number of patients with AF | ≥18 | Not specified |
| Record of died from cardiovascular cause during the measurement duration | Number of patients with AF | ≥18 | Not specified |
| Record of emotional well-being (including anxiety and depression) assessed using a validated instrument. | Number of patients with AF | ≥18 | Every visit |
| Record of haemorrhagic stroke during the measurement duration. | Number of patients with AF on anticoagulation | ≥18 | Not specified |
| Record of health-related quality of life assessed using a validated instrument. | Number of patients with AF | ≥18 | Every 12 months |
| Record of ischaemic stroke or transient ischaemic attack during the measurement duration | Number of patients with AF | ≥18 | Not specified |
| Record of left ventricular ejection fraction (LVEF)<40% and/or decompensated heart failure, and are inappropriately prescribed nondihydropyridine calcium-channel blocker | Number of patients with AF who have LVEF<40% and/or decompensated heart failure. | ≥18 | Not specified |
| Record of major procedural complications and/or drug-related serious adverse events during the measurement duration | Number of patients with AF on anticoagulation | ≥18 | Not specified |
| Record of modifiable risk factors (e.g. BP, obesity, obstructive sleep apnoea, alcohol excess, lack of exercise, poor glycaemic control, and smoking) identified | Number of patients with AF | ≥18 | Not specified |
| Record of offered cardioversion | Number of patients with new-onset persistent AF who are hemodynamically stable and in whom attempts to restore sinus rhythm were deemed appropriate | ≥18 | Not specified |
| Record of offered catheter ablation after the failure of, or intolerance to, one class I or class III antiarrhythmic drug | Number of patients with paroxysmal or persistent AF with no contraindications (or refusal) to catheter ablation who remain symptomatic on, or intolerant to, one class I or class III antiarrhythmic drug | ≥18 | Not specified |
| Record of patient engagement when deciding treatment strategy | Number of patients with AF | ≥18 | Not specified |
| Record of patient reported symptom status assessed using a validated instrument. | Number of patients with AF | ≥18 | Every 12 months |
| Record of patients with AF on anticoagulation who had documented life-threatening or major bleeding events during the measurement duration | Number of patients with AF on anticoagulation. | ≥18 | Not specified |
| Record of physical function assessed at the time of diagnosis using a validated instrument. | Number of patients with AF | ≥18 | Every visit |
| Record of serum creatinine checked | Number of patients with AF | ≥18 | Every visit |
| Record of therapeutic range (TTR) ≥70% for vitamin-K antagonist, and appropriate dose for non-vitamin K antagonist oral anticoagulation (NOAC) according to manufacturer recommendations. | Number of patients with AF on anticoagulation | ≥18 | Not specified |
| Record of unplanned hospitalisation for a cardiovascular cause during the measurement duration | Number of patients with AF | ≥18 | Not specified |
| Record of inappropriately prescribed dofetilide or sotalol | Number of patients with AF who have end-stage kidney disease, including patients on dialysis | ≥18 | Not specified |
| Patients with Atrial Fibrillation/Flutter and mitral valve stenosis or prosthetic heart valve that receive oral anticoagulation (warfarin (or other vitamin K antagonist). | Patients with Atrial Fibrillation/Flutter and mitral valve stenosis or prosthetic heart valve. | All | Every 12 months |
| Patients who have undergone catheter ablation and have a CHADS2 risk score of 2 or more without a contraindication who have a prescription for oral anticoagulation therapy (warfarin (or other vitamin K antagonist), dabigatran, rivaroxaban, apixaban) at one-year post-ablation. | Patients who have undergone catheter ablation of nonvalvular Atrial Fibrillation/Flutter and who have a  CHADS2score greater than or equal to 2 without a contraindication for oral anticoagulation who have at least one year of follow-up post-ablation | All | Every 12 months |
| Patients who have undergone repeat (1 or more) ablation for Atrial Fibrillation/Flutter within two years of first ablation. | Patients who have undergone catheter ablation of Atrial Fibrillation/Flutter with at least two years follow-up | All | Every 2 years |
| Patients with one or more of pre-specified major complication occurring post-catheter ablation of Atrial Fibrillation/Flutter within 30 days | Total number of patients who have undergone catheter ablation of Atrial Fibrillation/Flutter with at least 30 days follow-up. | All | 30 days |
| Patients who have a CHADS2 or CHA2DS2VASc Score documented on their medical record or have all of the risk criteria of these scores documented in their medical record | Patients with a newly diagnosis of nonvalvular atrial fibrillation/flutter | All | Not specified |
| Patients who have had an echocardiogram performed ± 6 months from date of their episode of newly diagnosed AF/AFL. | Patients with a newly diagnosed episode of AF/AFL. | All | Within 6 months |
| Patients in the denominator who are receiving an oral anticoagulant (warfarin [or other vitamin K antagonist], dabigatran, rivaroxaban, apixaban). | Patients with a diagnosis of nonvalvular atrial fibrillation/flutter who also meet the following inclusion criteria: 1) ≥75 years of age OR <75 years of age with a CHADS2 score ≥ 2, (in this instance either prior stroke/TIA/systemic embolus or at least two of hypertension, heart failure, or diabetes) 2) Without a contraindication for anticoagulation. 3) Patients alive at the end of first encounter from their qualifying episode of nonvalvular AF/AFL 4) Case selection time window to be determined at the time of analysis | All | Not specified |
| Patients who have a major haemorrhage | Patients with a qualifying episode of nonvalvular atrial fibrillation/flutter. | All | Not specified |
| Patients who suffer any stroke (excluding TIA). | Patients with a qualifying episode of nonvalvular atrial fibrillation/flutter. | All | Not specified |
| Patients with 12 or more INR measurements in a calendar year for patients with AF that are receiving Warfarin (or other vitamin K antagonist) and are more than 30 days from Qualifying AF diagnosis. | Patients with Atrial Fibrillation/Flutter that are receiving warfarin (or other vitamin K antagonist) and are more than 30 days for Qualifying AF diagnosis. | All | Every 12 months |
| Record of oral anticoagulation for atrial fibrillation | All patients with AF | All | Not specified |
| The number of patients whom stroke risk has been assessed using the CHA2DS2-VASc score risk stratification scoring system in the preceding 12 months. | The number of patients with atrial fibrillation. | All | Every 12 months |
| Record of ≥2 international normalised ratio testing of the patients who are on warfarin | Patients taking warfarin | All | Not specified |
| Record of INR in recommended range | All INR test done | All | Not specified |
| All patients with a diagnosis of nonvalvular atrial fibrillation/atrial flutter (NVAF/AFL) who have a CHADS2 or CHA2DS2VASc score or CHADS-65 or the elements of these scores (stroke/TIA/SE, hypertension, heart failure, age ≥ 75 y, diabetes, atherosclerotic disease, age 65-74 y, female sex) documented in their medical record | All patients with a diagnosis of NVAF/AFL | 65-74 | Every 12 months |
| Primary analysis: All patients with NVAF/AFL ≥75 y of age OR < 75 y of age and a CHADS2 score ≥2, and without a contraindication for OAC, who are receiving a prescription for an OAC (warfarin [or other VKA] apixaban, dabigatran, rivaroxaban) Secondary analysis: Include the possibility of reporting according to CHADS-65 and CHA2DS2VASc ≥2 in men and ≥3 in women | All patients with NVAF/AFL ≥ 75 y of age OR < 75 y of age and a CHADS2 score ≥2 | ≥20 | Every 12 months |
| Primary analysis: The number of patients with NVAF/AFL who are hospitalized for haemorrhage of any kind (an arbitrary definition of major bleeding) within a calendar year while taking an OAC  Secondary analysis: Possibility of reporting according to type of OAC (warfarin [or other VKA], apixaban, dabigatran, rivaroxaban | All patients with NVAF/AFL | ≥20 | Every 12 months |
| Patients with NVAF/AFL who are hospitalized for new HF | All patients with NVAF/AFL | ≥20 | Every 12 months |
| Patients with NVAF/AFL who have a stroke (within 1 y) | The number of patients with NVAF/AFL | ≥20 | Every 12 months |
| Assessment of stroke risk using the CHA2DS2-VASc score risk stratification scoring system (excluding those patients with a previous CHADS2 or CHA2DS2-VASc score of 2 or more) | Patients with atrial fibrillation (excluding those patients with a previous CHADS2 or CHA2DS2-VASc score of 2 or more) | < 75 | Every 12 months |
| Maintains a register of patients with atrial fibrillation | All practices | NA | Not specified |
| Record of a CHA2DS2-VASc score of 2 or more, the percentage of patients who are currently treated with anticoagulation drug therapy | Patients with atrial fibrillation | < 75 | Not specified |
| Recording of prescribed Warfarin or direct oral anticoagulant | All primary healthcare patients | ≥20 | Not specified |
| Recording of patients whose average international normalised ratio (INR) test results was 2-3 | All primary healthcare patients with atrial fibrillation on warfarin with ≥1 INR test result | ≥20 | Not specified |
| Recording of patients with diagnosis of atrial fibrillation/hospitalisation or emergency department visit for atrial fibrillation | All primary healthcare patients | ≥20 | Not specified |
| Prescription of anticoagulant medications to patients with atrial fibrillation and CHADS2 score ≥1 | All patients with atrial fibrillation | ≥18 | Not specified |
| **Mental health (n=25)** | | | |
| Women who have had an enquiry about their mental health between 4-16 weeks postpartum | Women who have given birth in the preceding 12 months | All | Every 12 months |
| Patients who have been reviewed within 10-35 days of the date of diagnosis. | Patients with a new diagnosis of depression | All | 10-35 days |
| Patients who have had a bio-psychosocial assessment by the point of diagnosis. | Patients with a new diagnosis of depression | All | Not specified |
| Patients in the denominator who have a record of BMI in the preceding 15 months. | Patients on the mental health register with a diagnosis of psychosis, schizophrenia or bipolar affective disease | All | Every 12 months |
| Patients who have a record of alcohol consumption in the preceding 15 months. | Patients on the mental health register with a diagnosis of psychosis, schizophrenia or bipolar affective disease. | All | Every 12 months |
| Patients who have a record of an offer of support and treatment for smoking within the preceding 12 months. | Patients with schizophrenia, bipolar affective disorder or other psychoses who are recorded as current smokers. | All | Every 12 months |
| Patients who have a record of blood glucose or HbA1c in the preceding 12 months. | Patients aged 18 and over on the mental health register with a diagnosis of psychosis, schizophrenia or bipolar affective disease. | >18 | Every 12 months |
| Patients who have a record of blood pressure in the preceding 15 months. | Patients on the mental health register with a diagnosis of psychosis, schizophrenia or bipolar affective disease. | All | Every 12 months |
| Patients who have a record of total cholesterol: HDL ratio in the preceding 12 months. | Patients aged 18 and over on the mental health register with a diagnosis of psychosis, schizophrenia or bipolar affective disease. | >18 | Every 12 months |
| Patients who have an agreed comprehensive care plan documented in their record (recorded in the preceding 12 months). | Patients with schizophrenia, bipolar disorder or other psychoses. | All | Every 12 months |
| Patients who have had a full formal cardiovascular disease risk assessment performed in the preceding 12 months. | Patients aged between 25 and 84 years with schizophrenia, bipolar disorder or other psychoses (excluding those with pre-existing cardiovascular disease, chronic kidney disease, familial hypercholesterolaemia or type 1 diabetes). | 25-84 | Every 12 months |
| Patients whose notes record smoking status in the preceding 12 months. | Patients registered with schizophrenia, bipolar affective disorder or other psychoses. | All | Every 12 months |
| Normal BP (150/90 mm Hg) in last 9 months | All hypertensive with SMI patients | ≥16 | Every 9 months |
| Record of last BP | All hypertensive with SMI patients | ≥16 | Every 9 months |
| Recording of Alcohol | All hypertensive with SMI patients | ≥16 | Not specified |
| Recording of BMI | All hypertensive with SMI patients | ≥16 | Not specified |
| Recording of BP | All hypertensive with SMI patients | ≥16 | Not specified |
| Recording of CHD comorbidity | All hypertensive with SMI patients | ≥16 | Not specified |
| Recording of cholesterol | All hypertensive with SMI patients | ≥16 | Not specified |
| Recording of CVD risk factor assessment | All hypertensive with SMI patients | ≥16 | Not specified |
| Recording of diabetes mellitus comorbidity | All hypertensive with SMI patients | ≥16 | Not specified |
| Recording of eGFR | All hypertensive with SMI patients | ≥16 | Not specified |
| Recording of HbA1c | All hypertensive with SMI patients | ≥16 | Not specified |
| Recording of smoking status | All hypertensive with SMI patients | ≥16 | Not specified |
| Recording of thyroid stimulating hormone | All hypertensive with SMI patients | ≥16 | Not specified |
| **Kidney disease (n=18)** | | | |
| Patients with type 2 diabetes and chronic kidney disease who are prescribed SGLT2 inhibitors | All patients with type 2 diabetes and chronic kidney disease | ≥18 | Not specified |
| Record of patients who have no contraindication, refusal, or history of intolerance to renin–angiotensin– aldosterone system inhibitors | All patients with diabetes and chronic kidney disease or hypertension | ≥18 | Not specified |
| Record of inappropriately prescribed dofetilide or sotalol | Number of patients with AF who have end-stage kidney disease, including patients on dialysis | ≥18 | Not specified |
| Record of a urine albumin: creatinine ratio (or protein: creatinine ratio) test | All CKD patients | All | Every 12 months |
| Record of last BP reading, measured is ≤140/85 mmHg | All CKD patients | All | Every 12 months |
| Record of BP in the register | All CKD patients | ≥18 | Every 12 months |
| Record of treated with angiotensin-converting enzyme inhibitor (ACE-I) or ARB | All patients with hypertension and proteinuria | ≥18 | Not specified |
| Record of 2 BP measures | All CKD patients | ≥18 | Not specified |
| Record of lipid profile | All CKD patients | ≥18 | Not specified |
| Record of recommended ACR test | All CKD patients | All | Not specified |
| Maintains a register of patients aged 18 years or over with CKD with classification of categories | All practices | >18 | Not specified |
| Recording of 2 BP reading | All CKD patients | >40 | Not specified |
| Recording of albumin to creatinine ratio | All CKD patients | >40 | Not specified |
| Recording of eGFR | All CKD patients | >40 | Not specified |
| Maintains a register of patients aged 18 or over with CKD with classification of categories | All practices | >18 | Not specified |
| Prescription of lipid-lowering medications | All CKD patients | ≥18 | Not specified |
| Achieved recommended target levels for BP<140/85 mmHg if diabetes and BP<130/80 mmHg if proteinuria | All patients with diabetes or proteinuria | All | Not specified |
| Prescribing an oral NSAID | All CKD patients | All | Not specified |
| **Dyslipidaemia (n=17)** | | | |
| Achievement of BP targets as per the guideline | All patients who were receiving at least one lipid-lowering treatment | ≥18 | Not specified |
| Achievement of total cholesterol targets (<200 mg/dl); LDL<130 mg/dl, HDL ≥40 mg/dl and triglyceride <200 mg/dl | All patients who were receiving at least one lipid-lowering treatment | ≥18 | Not specified |
| Patients with ≥1 cholesterol (total, LDL, HDL) assessment | All patients who were receiving at least one lipid-lowering treatment | >18 | Not specified |
| Patients' adherence to the prescribed statin regimen | All patients who were receiving at least one lipid-lowering treatment | ≥18 | Not specified |
| Patients for whom smoking status was registered | All patients who were receiving at least one lipid-lowering treatment | >18 | Not specified |
| Patients with ≥1 blood glucose test | All patients who were receiving at least one lipid-lowering treatment | >18 | Not specified |
| Patients with ≥1 BMI assessment | All patients who were receiving at least one lipid-lowering treatment | >18 | Not specified |
| Patients with ≥1 BP measurement | All patients who were receiving at least one lipid-lowering treatment | >18 | Not specified |
| Record of current use of lipid lowering medication (started before the date of blood test and did not stop up to three months before the time of the blood test) | All patients with dyslipidaemia | ≥18 | Not specified |
| Record of non-use of lipid lowering medication (no record of medication uses within two years of the lab test) | All patients with dyslipidaemia | ≥18 | Not specified |
| Record of previous use of lipid lowering medication | All patients with dyslipidaemia | ≥18 | Within 2 years |
| Record of lipid lowering medication prescribed | All patients with dyslipidaemia | ≥18 | Not specified |
| Record of lipid profile | All patients with dyslipidaemia | ≥18 | Not specified |
| Record of ≥1 encounter with prescription of statin therapy to patients with history of LDL >190 mg/dL, without diagnosis of ASCVD | All patients with dyslipidaemia | ≥18 | Every 12 months |
| Prescription of beta-blocker as a single agent | All hypertensive patients with T2DM or dyslipidaemia | ≥30 | Not specified |
| Prescription of diuretic as a single agent | All hypertensive patients with T2DM or dyslipidaemia | ≥30 | Not specified |
| Prescription of lipid-lowering medications | All patients with familial hypercholesterolaemia | ≥18 | Not specified |
| **Smoking (n=14)** | | | |
| Access to a smoking cessation program | All healthcare centres or units | NA | Not specified |
| Record of smoking cessation counselling | All smoker patients | ≥18 | Not specified |
| Record of smoking cessation drug prescribed | All smoker patients | ≥18 | Not specified |
| Record referral to smoking cessation program | All smoker patients | ≥18 | Not specified |
| Procedure for smoking cessation (e.g. Minimal Intervention Strategy) | All practices | NA | Not specified |
| The number of patients who have a record of an offer of support and treatment for smoking within the preceding 24 months. | The number of patients aged 15 years and over who are recorded as current smokers. | >15 | Every 2 years |
| Smoking status should be clearly identifiable from the paper and/or electronic record | All practices | NA | Not applicable |
| Offered ≥1 smoking cessation advice | All smokers | All | Not specified |
| Smoking—point abstinence (verified by carbon monoxide meter where CO > 8 ppm represents recent tobacco smoking) | All smoker patients | >18 | Every 12 months |
| Identified as tobacco user and received cessation counselling intervention | All tobacco user | ≥18 | Every 12 months |
| Recording of prescribed smoking cessation drug | All smokers | >40 | Not specified |
| Recording of provision of smoking program | All smokers | >40 | Not specified |
| Recording of smoking advice | All smokers | >40 | Not specified |
| Recording of received smoking cessation counselling | All primary healthcare patients who smoke | ≥20 | Not specified |
| **TIA (n=13)** | | | |
| Documented smoking status; Dietary advice; Exercise advice; BMI; Smokers have been given cessation advice; Overweight (BMI≥25) and obese (BMI≥30) patients have been given weight loss advice (dietary or exercise advice) | All TIA patients | ≥18 | Every 12 months |
| Record of antihypertensives are prescribed if BP >130/80: angiotensin-converting enzyme (ACE) inhibitor or, angiotensin receptor blocker (ARB) or calcium channel blocker (CCB) or thiazide-type diuretic | All TIA patients | ≥18 | Not specified |
| Record of antithrombotic are prescribed: aspirin and dipyridamole, aspirin alone, clopidogrel or anticoagulation | All TIA patients | ≥18 | Not specified |
| Record of BP | All TIA patients | ≥18 | Every 12 months |
| Record of combined risk factor control: most recent BP ≥130/80 mm Hg and most recent total cholesterol <4.0 mmol/l and most recent LDL <2.0 mmol/l and antithrombotic are prescribed | All TIA patients | ≥18 | Not specified |
| Record of combined secondary prevention medication: prescribed an antihypertensive, a statin and an antithrombotic | All TIA patients | ≥18 | Not specified |
| Record of LDL | All TIA patients | ≥18 | Every 12 months |
| Record of most recent BP ≤130/80 mm Hg | All TIA patients | ≥18 | Not specified |
| Record of most recent LDL <2 mmol/l | All TIA patients | ≥18 | Not specified |
| Record of most recent total cholesterol <4 mmol/l | All TIA patients | ≥18 | Not specified |
| Record of most recent total cholesterol <4.0 mmol/l or 25% reduction in total cholesterol, whichever achieves the lowest absolute value | All TIA patients | ≥18 | Not specified |
| Record of recent total cholesterol <4.0 mmol/l and most recent LDL <2.0 mmol/l | All TIA patients | ≥18 | Not specified |
| Record of statins are prescribed if total cholesterol >3.5 mmol/l or LDL >2.5 mmol/l (i.e., both measured) | All TIA patients | ≥18 | Not specified |
| **Heart failure (n=9)** | | | |
| Advice about quitting smoking | All heart failure patients | All | Not specified |
| Advice about the vaccination against influenza | All heart failure patients | All | Not specified |
| Smoking status | All heart failure patients | All | Not specified |
| Value of cholesterol≤5mmol/l | All heart failure patients | All | Every 12 months |
| Record of an offer of referral for an exercise-based rehabilitation programme | Patients with heart failure diagnosed | All | Every 15 months |
| Maintains a register of patients with heart failure | All practices | NA | Not specified |
| Record of heart failure confirmed by an echocardiogram or by specialist assessment 3 months before or 12 months after entering on to the register | Heart failure patients | < 75 | Before 3 months or After 12 months |
| the percentage of patients who are currently treated with an ACE-I or ARB | Patients with a current diagnosis of heart failure due to left ventricular systolic dysfunction | < 75 | Not specified |
| Treated with an ACE-I or ARB, the percentage of patients who  are additionally currently treated with a beta-blocker licensed for heart failure | Patients with a current diagnosis of heart failure due to left ventricular systolic dysfunction | < 75 | Not specified |
| **Other conditions (COPD, PAD, RHD, Peptic Ulcer) (n=9)** | | | |
| Patients with diagnosis of COPD AND who are immunised against influenza | All patients with COPD | >15 | Every 12 months |
| Maintains a register of patients with peripheral arterial disease. | All practices | NA | Not applicable |
| Patients with a record in the preceding 15 months that aspirin or an alternative antiplatelet is being taken. | The number of patients with peripheral arterial disease | All | Every 15 months |
| Practice has a smoking cessation program | Number of practices | NA | Not specified |
| Number of active patients with coronary artery disease (CAD) on anti-thrombotic. | Number of active patients with CAD | All | Not specified |
| Number of active patients with known coronary artery disease (CAD) prescribed statin or lipid lowering therapy | Number of active patients aged ≤ 80 years with known CAD | ≤ 80 | Not specified |
| Influenza vaccination | All patients with RHD or ARF | All | Every 24 months |
| Record of echo cardiogram | All patients with RHD or ARF | All | Every 3 years |
| Prescribing a traditional oral NSAID or low-dose aspirin | All patients with a history of peptic ulceration without co-prescription of gastro-protection | All | Not specified |
