# Supplementary material for: Quality indicators for the primary prevention of cardiovascular disease in primary care: A systematic review
Source: PLoS One. 2024 Dec 5;19(12):e0312137. doi: 10.1371/journal.pone.0312137 (PMC11620663; doi:10.1371/journal.pone.0312137)
Supplement: S10 Table — (DOCX) [file pone.0312137.s010.docx]

## S10 Table. Study characteristics extraction sheet

| **Author** | **Year** | **Country** | **World Bank category** | **Study design/Methodology** | **Study period** |
| --- | --- | --- | --- | --- | --- |
| Adeleke et. al. | 2019 | UK | High-income | Cohort study | 1 January 2011 - 31 September 2018 |
| Aktaa et. al. | 2022 | Multi-country | Upper middle-income | A Systematic review and Delphi method | 1 January 2016 - 8 March 2021 |
| Arbelo et. al. | 2021 | Multi-country | High-income | A Systematic review and Delphi method | 1 January 2014- 5 October 2019 |
| Arcoraci et. al. | 2014 | Italy | High-income | Cohort study | 1 June 2005 - 31 May 2008 |
| Aref-Eshghi et. al. | 2015 | Canada | High-income | Cross sectional study | 1 January 2009 - 31 December 2011 |
| Barlow & Krassas | 2013 | Australia | High-income | Cohort study | 2012 |
| Beganlic et. al. | 2015 | Bosnia and Herzegovina | Upper middle-income | Cross sectional study | 1 January 2012 - 31 December 2012 |
| Burgess et. al. | 2015 | Australia | High-income | Cross sectional study | 2012-2014 |
| Canadian Cardiovascular Society | 2019 | Canada | High-income | Grey literature | Not mentioned |
| Coleman et. al. | 2021 | United States | High-income | Randomised controlled trial | January 2015-January 2018 |
| Collins et. al. | 2021 | Tajikistan | Lower middle-income | Mixed methods | Not mentioned |
| Department of Health | 2020 | Australia | High-income | Grey literature | Not mentioned |
| deWet et. al. | 2012 | UK | High-income | Cohort study | February - April 2011 |
| Egan et. al. | 2018 | United States | High-income | Quasi experimental, pre- versus post study | 1 February 2015 - 31 May 2017 |
| Feng & Gravelle | 2019 | UK | High-income | Quality improvement study | 2005-2013 |
| Fleetcroft et. al. | 2012 | UK | High-income | Cross sectional study | 2008-2009 |
| Fonseca et. al. | 2022 | Portugal | High-income | Quality improvement study | December 2017- December 2018 |
| Furthauer et. al. | 2013 | Austria | High-income | Cross sectional study | January to April 2011 |
| Harris et. al. | 2012 | Australia | High-income | Randomised controlled trial | July 2008 - January 2010 |
| Harris et. al. | 2015 | Australia | High-income | Randomised controlled trial | 2012-2013 |
| Honeyford et. al. | 2013 | UK | High-income | Cross sectional study | April 2006 - March 2009 |
| Huber et. al. | 2020 | Switzerland | High-income | Cohort study | 2015-2016 |
| Karnad et. al. | 2018 | Multi-country | High-income | Cohort study | Not mentioned |
| Karunaratne et. al. | 2013 | UK | High-income | Cohort study | 1 April 2004 and 31 March 2010 |
| Khanji et. al. | 2019 | Canada | High-income | Randomised controlled trial (Secondary analysis) | Not mentioned |
| Knierim et. al. | 2019 | United States | High-income | Quality improvement study | February 2015-December 2017 |
| Knight et. al. | 2012 | Australia | High-income | Quality improvement study | Not mentioned |
| Kontopantelis et. al. | 2014 | UK | High-income | Cohort study | 2004/05 - 2011/12 |
| Lager et. al. | 2012 | UK | High-income | Cohort study | 1 February - 31 October 2009 |
| Liddy et. al. | 2012 | Canada | High-income | Cross sectional study | 2008-2010 |
| Lindner et. al. | 2019 | United States | High-income | Cross sectional study | September 2015-April 2017 |
| Ludt et. al. | 2013 | Multi-country | High-income | Cross sectional study | 2006-2010 |
| Ludt et. al. | 2014 | Multi-country | High-income | Cross sectional study | 2006-2010 |
| Naicker et. al. | 2014 | Canada | High-income | Cross sectional study | Not mentioned |
| NICE | 2023 | UK | High-income | Grey literature | Not mentioned |
| Novello et. al. | 2017 | Brasil | Upper middle-income | Cross sectional study | August 2011 and November 2012 |
| Persell et. al. | 2020 | United States | High-income | Randomised controlled trial | 8 February 2016 - 30 April, 2018 |
| Petek et. al. | 2012 | Slovenia | High-income | Literature Review & Modified Delphi process | 2011 |
| RACGP | 2015 | Australia | High-income | Grey literature | Not mentioned |
| Ralph et. al. | 2013 | Australia | High-income | Cross sectional study | January 2008 - December 2010 |
| Redfern et. al. | 2020 | Australia | High-income | Randomised controlled trial | Not mentioned |
| Sandhu et. al. | 2019 | Canada | High-income | Quality improvement study | 2017 |
| Schierhout et. al. | 2013 | Australia | High-income | Cross sectional study | April and December 2009 |
| Shah et. al. | 2013 | UK | High-income | Cohort study | September 2011 |
| Shelley et. al. | 2020 | United States | High-income | Randomised controlled trial | 1 January 2015 - 31 March 2018 |
| Singh et. al. | 2015 | Canada | High-income | Randomised control trial (secondary analysis) | Not mentioned |
| Szigethy et. al. | 2013 | Hungary | High-income | Cross sectional study | January - December 2011 |
| Teh et. al. | 2020 | Malaysia | Upper middle-income | Cross sectional study | 1 November 2016 - 30 June 2019 |
| The Health Foundation | 2015 | UK | High-income | Grey literature | Not mentioned |
| Tran et. al. | 2013 | Norway | High-income | Cross sectional study | 2003-2005 |
| Tu et. al. | 2017 | Canada | High-income | Literature Review & Modified Delphi process | 2012-2016 |
| Turner et. al. | 2016 | UK | High-income | Cohort study | 1 January 2009- 31 December 2013 |
| vanderPol et. al. | 2019 | UK | High-income | Cohort study | 1 April 2005 - 31 March 2012 |
| Webster et. al. | 2021 | Australia | High-income | Randomised controlled trial | December 2016 - September 2019 |
| Willis et. al. | 2017 | UK | High-income | Cross sectional study | 1 January 2012 - 31 March 2013 |
| Woodhead et. al. | 2016 | UK | High-income | Cohort study | 31 January 2012 - 31 October 2013 |
| Zhdan et. al. | 2017 | Ukraine | Lower middle-income | Cross sectional study | Not mentioned |
