# Supplementary material for: Quality indicators for the primary prevention of cardiovascular disease in primary care: A systematic review
Source: PLoS One. 2024 Dec 5;19(12):e0312137. doi: 10.1371/journal.pone.0312137 (PMC11620663; doi:10.1371/journal.pone.0312137)
Supplement: S11 Table — (DOCX) [file pone.0312137.s011.docx]

**S11 Table.** Summary of quality indicators reported in the articles as per Donabedian framework

| **Author/Year** | **STRUCTURE**  **INDICATOR** | **PROCESS INDICATOR** | | | | | | | **OUTCOME INDICATOR** |
| --- | --- | --- | --- | --- | --- | --- | --- | --- | --- |
|  | **Organisation of care** | **Assessment of risk factors** | **Global risk assessment** | **Record of lifestyle risk factors** | **Record of lifestyle advice** | **Prescriptions of medications** | **Referral** | **Risk communication/**  **advice** | **Attainment of risk factor targets** |
| Adeleke et al. , 2019 |  | X |  |  |  | X |  |  |  |
| Aktaa et.al., 2022 | X | X | X | X |  | X |  | X | X |
| Arbelo et. al., 2021 | X | X | X |  |  | X |  |  |  |
| Arcoraci et. al., 2014 |  | X |  | X |  | X |  |  | X |
| Aref-Eshghi et. al., 2015 |  |  |  | X |  | X |  |  | X |
| Barlow & Krassas, 2013 |  | X |  |  |  |  |  | X | X |
| Beganlic et. al., 2015 |  | X |  | X | X |  | X |  | X |
| Burgess et. al., 2015 |  |  | X |  |  | X |  |  | X |
| Canadian Cardiovascular Society 2019 | X | X |  |  |  | X |  |  |  |
| Coleman et. al., 2021 |  |  |  | X | X | X |  |  | X |
| Collins et. al., 2021 |  | X | X | X |  | X |  |  | X |
| de Wet et. al, 2012 |  | X |  | X | X | X |  |  | X |
| Department of Health, 2020 |  | X | X | X | X |  |  |  |  |
| Egan etl. al., 2018 | X | X |  | X |  | X |  |  | X |
| Feng & Gravelle, 2019 |  | X |  | X |  |  | X |  |  |
| Fleetcroft et. al., 2012 |  |  |  |  |  |  |  |  | X |
| Fonseca et. al., 2022 |  | X |  |  |  | X |  |  | X |
| Furthauer et. al., 2013 |  |  |  |  |  | X |  |  |  |
| Harris et. al., 2012 |  | X |  | X |  |  |  |  | X |
| Harris et. al., 2015 |  | X | X | X |  |  |  |  |  |
| Honeyford et.al., 2013 |  |  |  |  | X |  |  |  | X |
| Huber et. al., 2020 |  |  |  |  | X | X |  |  | X |
| Karnad et. al., 2018 |  | X |  |  |  |  |  |  |  |
| Karunaratne et. al., 2013 |  | X |  |  |  | X |  |  | X |
| Khanji et. al., 2019 | X | X | X | X | X | X |  | X | X |
| Knierim et al., 2019 |  |  |  |  | X | X |  |  | X |
| Knight et. al., 2012 | X |  |  |  |  |  |  | X | X |
| Kontopantelis et. al., 2014 |  | X |  | X | X |  |  |  | X |
| Lager et. al., 2012 |  | X |  | X |  | X |  |  | X |
| Liddy et. al., 2012 |  | X |  | X | X | X | X |  |  |
| Linder et. al., 2019 |  | X |  |  | X | X |  |  | X |
| Ludt et. al., 2013 | X | X |  | X | X |  |  |  | X |
| Ludt et. al., 2014 |  | X |  | X | X |  |  | X | X |
| Naicker et. al., 2014 |  | X |  | X | X | X | X |  |  |
| NICE 2023 | X | X | X | X | X | X | X | X |  |
| Novello et. al., 2017 |  |  |  | X |  | X |  |  | X |
| Persell et. al., 2020 |  |  |  |  | X |  |  |  | X |
| Petek et al. , 2012 | X | X | X | X | X | X |  | X | X |
| RACGP, 2015 | X | X | X | X |  | X |  |  |  |
| Ralph et. al., 2013 |  | X |  | X | X |  |  |  | X |
| Redfern et. al., 2020 |  | X |  | X |  |  |  |  | X |
| Sandhu et. al., 2019 |  | X |  |  |  | X |  |  |  |
| Schierhout et. al., 2013 |  | X |  | X |  | X |  |  | X |
| Shah et. al., 2013 |  | X |  |  | X | X |  |  |  |
| Shelley et. al., 2020 |  |  |  |  | X | X |  |  | X |
| Singh et. al., 2015 | X | X |  | X | X | X |  |  |  |
| Szigethy et. al., 2013 |  |  |  |  |  |  |  |  | X |
| Teh et. al., 2020 |  | X | X | X | X | X |  |  | X |
| The Health Foundation 2015 | X | X |  | X | X | X | X |  | X |
| Tran et. al., 2013 |  |  |  |  |  | X |  |  | X |
| Tu et. al., 2017 |  | X |  | X | X | X |  |  | X |
| Turner et.al., 2016 |  |  |  |  |  | X |  |  |  |
| VanderPol et. al., 2019 |  | X |  |  | X |  |  |  | X |
| Webster et.al., 2021 |  |  | X |  |  | X |  |  | X |
| Willis et al., 2017 |  | X | X |  |  | X |  |  | X |
| Woodhead et.al., 2016 |  | X | X | X |  |  |  |  | X |
| Zhdan et. al., 2017 |  | X |  |  |  |  |  |  |  |

RACGP, Royal Australian College of General Practitioners; NICE, National Institute for Health and Care Excellence.
