# Supplementary material for: Quality indicators for the primary prevention of cardiovascular disease in primary care: A systematic review
Source: PLoS One. 2024 Dec 5;19(12):e0312137. doi: 10.1371/journal.pone.0312137 (PMC11620663; doi:10.1371/journal.pone.0312137)
Supplement: Attachment — Submitted filename: Response to Editor and Reviewers.docx [file pone.0312137.s013.docx]

**Response to editor and reviewers**

**Title:** Quality indicators for the primary prevention of cardiovascular disease in primary care: A systematic review

**Manuscript ID:** PONE-D-24-16110

We thank the editors and reviewers for their time to review the manuscript and provide constructive comments. We have addressed these comments with each change outlined in the point-by-point response below and in the tracked version of the revised manuscript.

Please note: Editor and reviewers’ comments are shown in **bold** and page numbers as well as line numbers refer to the clean version of the manuscript*.* Yellow highlights are also used in certain situations below to indicate where a specific change has been made within the manuscript.

**EDITOR COMMENTS**

**1) Please ensure that your manuscript meets PLOS ONE's style requirements, including those for file naming. The PLOS ONE style templates can be found at**
<https://journals.plos.org/plosone/s/file?id=wjVg/PLOSOne_formatting_sample_main_body.pdf> **and**
<https://journals.plos.org/plosone/s/file?id=ba62/PLOSOne_formatting_sample_title_authors_affiliations.pdf>

**Response:** We have carefully reviewed the PLOS ONE's style requirements and have further aligned the article editing style including size of headings, table and figure citations and references. We have also confirmed that the files are named appropriately. Further, the figures are now uploaded in the appropriate format (TIF) and the Supporting Information is provided in pdf format.

**2) We note that the grant information you provided in the ‘Funding Information’ and ‘Financial Disclosure’ sections do not match. When you resubmit, please ensure that you provide the correct grant numbers for the awards you received for your study in the ‘Funding Information’ section.**

**Response:** Thank you for this comment. We have further confirmed and refined, as copied below:

**Funding**

Kiran Bam received Monash International Tuition Scholarship and Monash Graduate Scholarship support from Monash University, Melbourne, Victoria, Australia. Dominique A Cadilhac and Monique F Kilkenny received research fellowship support from the National Health and Medical Research Council of Australia (DAC: 1154273, MFK:1141848). Monique F Kilkenny received fellowship support from the National Heart Foundation of Australia (MFK: 105737).

**Disclosures**

Dominique A Cadilhac received restricted grants from Boehringer Ingelheim, Moleac, Amazon Web Services, Nicolab, Philips and Medtronic; outside the submitted work. This does not alter our adherence to PLOS ONE policies on sharing data and materials.

**3) Thank you for stating the following in the Competing Interests section:**

**[DAC received restricted grants from Boehringer Ingelheim, Bristol-Myers, Moleac and Medtronic; and DAC and MFK from Amgen, outside the submitted work.].**

Please confirm that this does not alter your adherence to all PLOS ONE policies on sharing data and materials, by including the following statement: "This does not alter our adherence to PLOS ONE policies on sharing data and materials.” (as detailed online in our guide for authors <http://journals.plos.org/plosone/s/competing-interests>).  If there are restrictions on sharing of data and/or materials, please state these. Please note that we cannot proceed with consideration of your article until this information has been declared.

**Response:** We have now updated the conflict of interest form and have now added the following statement to confirm that this will not alter the data sharing policy of PLOS ONE.

"This does not alter our adherence to PLOS ONE policies on sharing data and materials.”

**4) Please include your updated Competing Interests statement in your cover letter; we will change the online submission form on your behalf."**

**Response:** We have updated Competing Interests in cover letter and statement in Page 16, Lines 545-546, as copied below:

The authors declare no competing interests exist.

**5) Please remove your figures from within your manuscript file, leaving only the individual TIFF/EPS image files, uploaded separately. These will be automatically included in the reviewers’ PDF.**

**Response:** We have ensured that all the figures are now removed from the manuscript file and all the individual figures in TIFF format adjusted through Preflight Analysis and Conversion Engine (PACE) are uploaded separately.

**6) While revising your submission, please upload your figure files to the Preflight Analysis and Conversion Engine (PACE) digital diagnostic tool,** [**https://pacev2.apexcovantage.com/**](https://pacev2.apexcovantage.com/)**. PACE helps ensure that figures meet PLOS requirements. To use PACE, you must first register as a user. Registration is free. Then, login and navigate to the UPLOAD tab, where you will find detailed instructions on how to use the tool. If you encounter any issues or have any questions when using PACE, please email PLOS at** [**figures@plos.org**](mailto:figures@plos.org)**. Please note that Supporting Information files do not need this step.**

**Response:** We have ensured that all the figures meet PLOS requirements using the Preflight Analysis and Conversion Engine (PACE) digital diagnostic tool and uploaded PACE adjusted images.

**REVIEWERS COMMENTS**

**A) Reviewer #1:**

**1) Thank you for this study. The public health case for this study is clearly necessary.**

**Response:** Thank you so much. We appreciate your positive response.

**2) How was the screening done? It may be useful to include information on the process of screening the articles. Typically, the articles will first be screened by title, then abstract, and finally full-text screening (this information was sort of provided under the strengths and limitations, but it would be helpful to clarify this under methods). The authors did a good job by providing the respective role of the reviewers, but they stop short of detailing the screening process. Also, were any of the articles eliminated using automated tools?**

**Response:** Thank you for this comment. We have now elaborated “Article screening” under “Methods” section in Page 4-5, Lines 158-170, as copied below. Further, we have also clarified that no automated tools were used to eliminate the articles, except for eliminating duplicates through Endnote version 19 or Covidence.

## *Article screening*

Articles obtained from the literature search were managed in EndNote version 19. The duplicates were identified and removed through EndNote using “find duplicates” feature. Manual review was also performed to check for any remaining duplicates, and then imported into Covidence for screening and data extraction [16]. No other automated tools were used to eliminate the articles. KB, MFK and MTO screened the titles and abstracts of articles for eligibility. At this stage, we excluded articles that were not related to quality indicators for cardiovascular disease or stroke based on title and abstract. Any conflicts between reviewers were resolved together by KB, MFK and MTO. To facilitate full-text screening, a review checklist **(S3 Table)** was developed to check against eligibility criteria**.** Full-text screening was done by KB, MFK and MTO. Disagreements between any two authors were adjudicated by DAC. At this stage, the primary reason for exclusion was recorded and summarised in the PRISMA flowchart **(Fig 1).**

**3) Authors should justify the tools, particularly the critical appraisal tool and framework for assessing the quality indicators that were used.**

**Response 1:** We have further elaborated on use of frameworks or tools used for assessing the quality indicators in “Methods” section in Page 5, Lines 171-196, as copied below:

## *Data extraction and synthesis*

The excel file template used for data extraction was initially developed by KB, MFK, MTO, and DAC, with inputs from the advisory group (other co-authors), and modified as the extraction progressed. The final data extraction table for study characteristics included information for author, year of publication, country, study design/methodology, and study period **(S4 Table)**. Other important information collected on summary of quality indicators include condition, domains, theme, type of indicator, age group (years), numerator, denominator, and the frequency of collection **(S5 Table)**. Data extraction was undertaken by KB, reviewed by MTO, and any disagreements were adjudicated by MFK or DAC.

Details about the quality indicators including numerator, denominator, age group (in years) and frequency of collection were extracted and summarised in tabular format **(S5 Table)**. For further data synthesis, themes and conditions were identified based on literature [14, 17-21], pre-tested across the initial set of the articles. For categorisation of domains and type of indicator, a theory informed conceptual framework **(Fig 2)** was developed from existing literature [14, 18, 20-23], and the Donabedian’s multidimensional framework [24, 25] of structure, process and outcome was used. This framework facilitated a comprehensive summary and evaluation of care quality across domains of primary care services [26], as described below.

## a) **Structure indicators** include infrastructure or human resources-related indicators that designate the conditions under which primary care is provided (*organisation of care*);

## b) **Process indicators** include the actions and processes of clinical care delivered by primary care physician, such as screening, ordering diagnostic tests, prescribing treatment i.e. medications, or monitoring (*assessment of metabolic risk factors, global risk assessment, lifestyle management, prescription of medications, risk communication/advice, or referral*); and

## c) **Outcome indicators** reflect changes in individuals resulting from the intervention provided at the primary care (*attainment of risk factor targets*).

**Response 2:** We have now elaborated on the critical appraisal tool for assessing the quality indicators in “Methods” section in Page 6, Lines 197-207, as copied below:

***Methodological quality appraisal of the indicators in the articles (including grey literature)***

Quality appraisal of the indicators reported in the articles and their development process was undertaken using the Appraisal of Indicators through Research and Evaluation (AIRE) instrument [27, 28] **(S6 Table).** The AIRE instrument is a validated and reliable tool to appraise the methodological quality of indicators [29]. The AIRE instrument is used by researchers, evaluators, and policymakers to enable the standardised assessment of the robustness and relevance of indicators [30]. The tool was developed from the Appraisal of Guidelines through Research and Evaluation (AGREE) instrument [31], a commonly used instrument to assess the methodological rigor. The AIRE has been used previously in systematic reviews in various settings [32-36].

**4) Authors should consider providing a sample of the search terms, preferably as a search string. This bolsters the methodological integrity of the study and promotes its reproducibility.**

**Response**: We agree with the reviewer, and have now added the key search terms to the “Methods” section in Page 3-4, Lines 125-150, as copied below:

The search terms used were “cardiovascular disease” or “stroke” AND “risk factors” AND “quality indicators” AND “primary care” AND “prevention”. Boolean operators such as AND, and OR were used to combine these search terms. The search terms were identified based on the initial scoping searches of the literature in Google Scholar and refined based on five relevant articles. Truncation (e.g., cardiovasc*, cerebrovasc*, stroke*) and Wildcards (e.g., cardiovasc$, cerebrovasc$) were used to broaden results and capture variations of words. We searched each database using a range of Medical Subject Headings (MeSH) terms to find all potentially relevant articles.

Below is an example of the search terms used in CINAHL Plus:

(Cardiovasc* adj1 disease* OR heart adj1 disease* OR Stroke* OR Transient isch*or Haemorrhag* adj1 stroke OR hemorrhag* adj1 stroke OR TIA OR myocardial infarction OR Cerebrovasc* event* OR Cerebrovasc* disease* OR Cerebrovasc* accident* OR cardiac failure) AND (Risk* OR Hypertension OR High adj2 pressure OR Dyslipi* OR Hyperlipi* OR atrial adj1 fibrillation OR Smoking OR Diabetes OR diabetes mellitus OR Alcohol OR Physical adj2 activity OR Obesity OR Mental health OR mental disorder* OR depress* OR anxiety OR psychiat* OR well-being OR quality of life OR self esteem OR self perception) AND (Quality adj1 care* OR Quality assessment OR Quality indicator* OR Quality assurance OR medication* OR medicine* Quality improvement OR Quality tool* OR Quality monitor* OR Quality metric OR Quality criter* OR Performance indicator OR Process adj2 care OR Performance measure OR Benchmark OR benchmarking OR Outcome measure OR Outcome indicator* OR Consensus OR delphi) AND (Primary adj2 care OR General adj1 practi* OR general practice OR general practitioners OR Clinical practice OR Family practice OR primary adj2 physician OR healthcare delivery) AND (primary adj2 prevention OR secondary adj2 prevention OR prevention OR control)

The summary of the different databases and full search strategy is documented in **S1 Table,** as copied below.

**S1 Table.** Overview of search strategy

| Database | Search strategy |
| --- | --- |
| Ovid Medline | 1 (Cardiovasc* adj1 disease*).tw.  2 (heart adj1 disease*).tw.  3 myocardial infarction/  4 Cerebrovasc* event*.tw.  5 Cerebrovasc* disease*.tw.  6 Cerebrovasc* accident*.tw.  7 (Cardiac adj1 failure).tw.  8 Stroke*.tw. or stroke/  9 Transient isch*.tw.  10 TIA.tw.  11 (Haemorrhag* adj1 stroke).tw.  12 (hemorrhag* adj1 stroke).tw.  13 1 or 2 or 3 or 4 or 5 or 6 or 7 or 8 or 9 or 10 or 11 or 12  14 Risk*.tw.  15 Hypertension.tw.  16 (High adj2 pressure).tw.  17 Dyslipi*.tw.  18 Hyperlipi*.tw.  19 Smoking.tw.  20 atrial fibrillation.tw.  21 Diabetes.tw. or diabetes mellitus/  22 Alcohol.tw.  23 (Physical adj2 activity).tw.  24 Obesity.tw.  25 ((Mental adj1 health) or (mental adj1 disorder*) or depress* or anxiety or psychiat* or psycho* or well-being or (quality adj1 life) or (self adj1 esteem) or (self adj1 perception)).tw.  26 14 or 15 or 16 or 17 or 18 or 19 or 20 or 21 or 22 or 23 or 24 or 25  27 (primary adj2 prevention).tw.  28 (secondary adj2 prevention).tw.  29 (Prevention or control).tw.  30 27 or 28 or 29  31 (Quality adj1 care*).tw.  32 Quality assessment.tw.  33 Quality indicator*.tw.  34 Quality assurance.tw.  35 (medication* or medicine*).tw.  36 Quality improvement.tw.  37 Quality tool*.tw.  38 Quality monitor*.tw.  39 Quality metric.tw.  40 Quality criter*.tw.  41 Performance indicator.tw.  42 (Process adj2 care).tw.  43 Performance measure.tw.  44 Benchmark.tw. or benchmarking/  45 Outcome measure.tw.  46 Outcome indicator*.tw.  47 (Consensus or delphi).tw.  48 31 or 32 or 33 or 34 or 35 or 36 or 37 or 38 or 39 or 41 or 42 or 43 or 44 or 45 or 46 or 47  49 (Primary adj2 care).tw.  50 (General adj1 practi*).tw. or exp general practice/ or general practitioners/  51 Clinical practice.tw.  52 Family practice.tw.  53 (primary adj2 physician).tw.  54 healthcare delivery.tw.  55 49 or 50 or 51 or 52 or 53 or 54  56 13 and 26 and 30 and 48 and 55  57 limit 56 to (english language and yr="2012 -Current") |
| Ovid EMBASE | 1 (Cardiovasc* adj1 disease*).tw.  2 (heart adj1 disease*).tw.  3 myocardial infarction/  4 Cerebrovasc* event*.tw.  5 Cerebrovasc* disease*.tw.  6 Cerebrovasc* accident*.tw.  7 (Cardiac adj1 failure).tw.  8 Stroke*.tw. or stroke/  9 Transient isch*.tw.  10 TIA.tw.  11 (Haemorrhag* adj1 stroke).tw.  12 (hemorrhag* adj1 stroke).tw.  13 1 or 2 or 3 or 4 or 5 or 6 or 7 or 8 or 9 or 10 or 11 or 12  14 Risk*.tw.  15 Hypertension.tw.  16 (High adj2 pressure).tw.  17 Dyslipi*.tw.  18 Hyperlipi*.tw.  19 Smoking.tw.  20 atrial fibrillation.tw.  21 Diabetes.tw. or diabetes mellitus/  22 Alcohol.tw.  23 (Physical adj2 activity).tw.  24 Obesity.tw.  25 ((Mental adj1 health) or (mental adj1 disorder*) or depress* or anxiety or psychiat* or psycho* or well-being or (quality adj1 life) or (self adj1 esteem) or (self adj1 perception)).tw.  26 14 or 15 or 16 or 17 or 18 or 19 or 20 or 21 or 22 or 23 or 24 or 25  27 (primary adj2 prevention).tw.  28 (secondary adj2 prevention).tw.  29 (Prevention or control).tw.  30 27 or 28 or 29  31 (Quality adj1 care*).tw.  32 Quality assessment.tw.  33 Quality indicator*.tw.  34 Quality assurance.tw.  35 (medication* or medicine*).tw.  36 Quality improvement.tw.  37 Quality tool*.tw.  38 Quality monitor*.tw.  39 Quality metric.tw.  40 Quality criter*.tw.  41 Performance indicator.tw.  42 (Process adj2 care).tw.  43 Performance measure.tw.  44 Benchmark.tw. or benchmarking/  45 Outcome measure.tw.  46 Outcome indicator*.tw.  47 (Consensus or delphi).tw.  48 31 or 32 or 33 or 34 or 35 or 36 or 37 or 38 or 39 or 41 or 42 or 43 or 44 or 45 or 46 or 47  49 (Primary adj2 care).tw.  50 (General adj1 practi*).tw. or exp general practice/ or general practitioners/  51 Clinical practice.tw.  52 Family practice.tw.  53 (primary adj2 physician).tw.  54 healthcare delivery.tw.  55 49 or 50 or 51 or 52 or 53 or 54  56 13 and 26 and 30 and 48 and 55  57 limit 56 to (english language and yr="2012 -Current")  58 limit 57 to conference abstracts  59 57 not 58 |
| CINAHL Plus | (Cardiovasc* adj1 disease* OR heart adj1 disease* OR Stroke* OR Transient isch*or Haemorrhag* adj1 stroke OR hemorrhag* adj1 stroke OR TIA OR myocardial infarction OR Cerebrovasc* event* OR Cerebrovasc* disease* OR Cerebrovasc* accident* OR cardiac failure) AND (Risk* OR Hypertension OR High adj2 pressure OR Dyslipi* OR Hyperlipi* OR atrial adj1 fibrillation OR Smoking OR Diabetes OR diabetes mellitus OR Alcohol OR Physical adj2 activity OR Obesity OR Mental health OR mental disorder* OR depress* OR anxiety OR psychiat* OR well-being OR quality of life OR self esteem OR self perception) AND (Quality adj1 care* OR Quality assessment OR Quality indicator* OR Quality assurance OR medication* OR medicine* Quality improvement OR Quality tool* OR Quality monitor* OR Quality metric OR Quality criter* OR Performance indicator OR Process adj2 care OR Performance measure OR Benchmark OR benchmarking OR Outcome measure OR Outcome indicator* OR Consensus OR delphi) AND (Primary adj2 care OR General adj1 practi* OR general practice OR general practitioners OR Clinical practice OR Family practice OR primary adj2 physician OR healthcare delivery) AND (primary adj2 prevention OR secondary adj2 prevention OR prevention OR control) |
| SCOPUS | TITLE-ABS-KEY ("cardiovasc$ PRE/1 disease$" OR "heart PRE/1 disease$" OR "myocardial infarction" OR "cerebrovasc$ event$" OR "cerebrovasc$ disease$" OR "cerebrovasc$ accident$" OR "cardiac failure") OR ("stroke" OR "transient isch$" OR "haemorrhag$ PRE/1 stroke" OR "hemorrhag$ PRE/1 stroke") AND TITLE-ABS-KEY ("Risk$" OR "Hypertension" OR "High PRE/2 pressure" OR "Dyslipi$" OR "Hyperlipi$" OR "Smoking" OR "Diabetes" OR "diabetes mellitus" OR "Alcohol" OR "Physical PRE/2 activity" OR "Obesit$" OR "mental health" OR "mental disorder" OR "depress$" OR "anxiety" OR "psycho$" OR "psychiat$" OR "well-being" OR "quality of life" OR "self esteem" OR "self perception") AND TITLE-ABS-KEY ("Quality PRE/1 care$" or "Quality assessment" or "Quality indicator$" or "Quality assurance" or "medication$" or "medicine$" "Quality improvement" or "Quality tool$" or "Quality monitor$" or "Quality metric" or "Quality criter$" or "Performance indicator" or "Process PRE/2 care" or "Performance measure" or "Benchmark" or "benchmarking" or "Outcome measure" or "Outcome indicator$" or "Consensus" or "delphi") AND TITLE-ABS-KEY ("Primary PRE/2 care" or "General PRE/1 practi$" or "general practice" or "general practitioners" or "Clinical practice" or "Family practice" or "primary PRE/2 physician" or "healthcare delivery") AND TITLE-ABS-KEY ("primary PRE/2 prevention" OR "secondary PRE/2 prevention" OR "Prevention" OR "Control") AND ( EXCLUDE ( DOCTYPE,"cp" ) OR EXCLUDE ( DOCTYPE,"ed" ) ) AND ( LIMIT-TO ( PUBYEAR,2022) OR LIMIT-TO ( PUBYEAR,2021) OR LIMIT-TO ( PUBYEAR,2020) OR LIMIT-TO ( PUBYEAR,2019) OR LIMIT-TO ( PUBYEAR,2018) OR LIMIT-TO ( PUBYEAR,2017) OR LIMIT-TO ( PUBYEAR,2016) OR LIMIT-TO ( PUBYEAR,2015) OR LIMIT-TO ( PUBYEAR,2014) OR LIMIT-TO ( PUBYEAR,2013) OR LIMIT-TO ( PUBYEAR,2012) ) AND ( LIMIT-TO ( LANGUAGE,"English" ) ) |

**5) Given that the concept of the review is clinical in nature, the authors may want to check the search terms in MeSH to ensure that they captured all the possible variations of the search terms.**

**AND**

**Response:** Thank you for this comment. We developed our search strategy based on the medical and subject headings (MeSH) terms, and adjusted the terms slightly to conform to the format of the different databases. We have now clarified our approach in the Methods section (Page 4, Lines 131-134), as copied below:

We searched each database using a range of Medical Subject Headings (MeSH) terms to find all potentially relevant articles. The summary of the different databases and full search strategy is documented in **S1 Table** as copied below:

**S1 Table.** Overview of search strategy

| Database | Search strategy |
| --- | --- |
| Ovid Medline | 1 (Cardiovasc* adj1 disease*).tw.  2 (heart adj1 disease*).tw.  3 myocardial infarction/  4 Cerebrovasc* event*.tw.  5 Cerebrovasc* disease*.tw.  6 Cerebrovasc* accident*.tw.  7 (Cardiac adj1 failure).tw.  8 Stroke*.tw. or stroke/  9 Transient isch*.tw.  10 TIA.tw.  11 (Haemorrhag* adj1 stroke).tw.  12 (hemorrhag* adj1 stroke).tw.  13 1 or 2 or 3 or 4 or 5 or 6 or 7 or 8 or 9 or 10 or 11 or 12  14 Risk*.tw.  15 Hypertension.tw.  16 (High adj2 pressure).tw.  17 Dyslipi*.tw.  18 Hyperlipi*.tw.  19 Smoking.tw.  20 atrial fibrillation.tw.  21 Diabetes.tw. or diabetes mellitus/  22 Alcohol.tw.  23 (Physical adj2 activity).tw.  24 Obesity.tw.  25 ((Mental adj1 health) or (mental adj1 disorder*) or depress* or anxiety or psychiat* or psycho* or well-being or (quality adj1 life) or (self adj1 esteem) or (self adj1 perception)).tw.  26 14 or 15 or 16 or 17 or 18 or 19 or 20 or 21 or 22 or 23 or 24 or 25  27 (primary adj2 prevention).tw.  28 (secondary adj2 prevention).tw.  29 (Prevention or control).tw.  30 27 or 28 or 29  31 (Quality adj1 care*).tw.  32 Quality assessment.tw.  33 Quality indicator*.tw.  34 Quality assurance.tw.  35 (medication* or medicine*).tw.  36 Quality improvement.tw.  37 Quality tool*.tw.  38 Quality monitor*.tw.  39 Quality metric.tw.  40 Quality criter*.tw.  41 Performance indicator.tw.  42 (Process adj2 care).tw.  43 Performance measure.tw.  44 Benchmark.tw. or benchmarking/  45 Outcome measure.tw.  46 Outcome indicator*.tw.  47 (Consensus or delphi).tw.  48 31 or 32 or 33 or 34 or 35 or 36 or 37 or 38 or 39 or 41 or 42 or 43 or 44 or 45 or 46 or 47  49 (Primary adj2 care).tw.  50 (General adj1 practi*).tw. or exp general practice/ or general practitioners/  51 Clinical practice.tw.  52 Family practice.tw.  53 (primary adj2 physician).tw.  54 healthcare delivery.tw.  55 49 or 50 or 51 or 52 or 53 or 54  56 13 and 26 and 30 and 48 and 55  57 limit 56 to (english language and yr="2012 -Current") |
| Ovid EMBASE | 1 (Cardiovasc* adj1 disease*).tw.  2 (heart adj1 disease*).tw.  3 myocardial infarction/  4 Cerebrovasc* event*.tw.  5 Cerebrovasc* disease*.tw.  6 Cerebrovasc* accident*.tw.  7 (Cardiac adj1 failure).tw.  8 Stroke*.tw. or stroke/  9 Transient isch*.tw.  10 TIA.tw.  11 (Haemorrhag* adj1 stroke).tw.  12 (hemorrhag* adj1 stroke).tw.  13 1 or 2 or 3 or 4 or 5 or 6 or 7 or 8 or 9 or 10 or 11 or 12  14 Risk*.tw.  15 Hypertension.tw.  16 (High adj2 pressure).tw.  17 Dyslipi*.tw.  18 Hyperlipi*.tw.  19 Smoking.tw.  20 atrial fibrillation.tw.  21 Diabetes.tw. or diabetes mellitus/  22 Alcohol.tw.  23 (Physical adj2 activity).tw.  24 Obesity.tw.  25 ((Mental adj1 health) or (mental adj1 disorder*) or depress* or anxiety or psychiat* or psycho* or well-being or (quality adj1 life) or (self adj1 esteem) or (self adj1 perception)).tw.  26 14 or 15 or 16 or 17 or 18 or 19 or 20 or 21 or 22 or 23 or 24 or 25  27 (primary adj2 prevention).tw.  28 (secondary adj2 prevention).tw.  29 (Prevention or control).tw.  30 27 or 28 or 29  31 (Quality adj1 care*).tw.  32 Quality assessment.tw.  33 Quality indicator*.tw.  34 Quality assurance.tw.  35 (medication* or medicine*).tw.  36 Quality improvement.tw.  37 Quality tool*.tw.  38 Quality monitor*.tw.  39 Quality metric.tw.  40 Quality criter*.tw.  41 Performance indicator.tw.  42 (Process adj2 care).tw.  43 Performance measure.tw.  44 Benchmark.tw. or benchmarking/  45 Outcome measure.tw.  46 Outcome indicator*.tw.  47 (Consensus or delphi).tw.  48 31 or 32 or 33 or 34 or 35 or 36 or 37 or 38 or 39 or 41 or 42 or 43 or 44 or 45 or 46 or 47  49 (Primary adj2 care).tw.  50 (General adj1 practi*).tw. or exp general practice/ or general practitioners/  51 Clinical practice.tw.  52 Family practice.tw.  53 (primary adj2 physician).tw.  54 healthcare delivery.tw.  55 49 or 50 or 51 or 52 or 53 or 54  56 13 and 26 and 30 and 48 and 55  57 limit 56 to (english language and yr="2012 -Current")  58 limit 57 to conference abstracts  59 57 not 58 |
| CINAHL Plus | (Cardiovasc* adj1 disease* OR heart adj1 disease* OR Stroke* OR Transient isch*or Haemorrhag* adj1 stroke OR hemorrhag* adj1 stroke OR TIA OR myocardial infarction OR Cerebrovasc* event* OR Cerebrovasc* disease* OR Cerebrovasc* accident* OR cardiac failure) AND (Risk* OR Hypertension OR High adj2 pressure OR Dyslipi* OR Hyperlipi* OR atrial adj1 fibrillation OR Smoking OR Diabetes OR diabetes mellitus OR Alcohol OR Physical adj2 activity OR Obesity OR Mental health OR mental disorder* OR depress* OR anxiety OR psychiat* OR well-being OR quality of life OR self esteem OR self perception) AND (Quality adj1 care* OR Quality assessment OR Quality indicator* OR Quality assurance OR medication* OR medicine* Quality improvement OR Quality tool* OR Quality monitor* OR Quality metric OR Quality criter* OR Performance indicator OR Process adj2 care OR Performance measure OR Benchmark OR benchmarking OR Outcome measure OR Outcome indicator* OR Consensus OR delphi) AND (Primary adj2 care OR General adj1 practi* OR general practice OR general practitioners OR Clinical practice OR Family practice OR primary adj2 physician OR healthcare delivery) AND (primary adj2 prevention OR secondary adj2 prevention OR prevention OR control) |
| SCOPUS | TITLE-ABS-KEY ("cardiovasc$ PRE/1 disease$" OR "heart PRE/1 disease$" OR "myocardial infarction" OR "cerebrovasc$ event$" OR "cerebrovasc$ disease$" OR "cerebrovasc$ accident$" OR "cardiac failure") OR ("stroke" OR "transient isch$" OR "haemorrhag$ PRE/1 stroke" OR "hemorrhag$ PRE/1 stroke") AND TITLE-ABS-KEY ("Risk$" OR "Hypertension" OR "High PRE/2 pressure" OR "Dyslipi$" OR "Hyperlipi$" OR "Smoking" OR "Diabetes" OR "diabetes mellitus" OR "Alcohol" OR "Physical PRE/2 activity" OR "Obesit$" OR "mental health" OR "mental disorder" OR "depress$" OR "anxiety" OR "psycho$" OR "psychiat$" OR "well-being" OR "quality of life" OR "self esteem" OR "self perception") AND TITLE-ABS-KEY ("Quality PRE/1 care$" or "Quality assessment" or "Quality indicator$" or "Quality assurance" or "medication$" or "medicine$" "Quality improvement" or "Quality tool$" or "Quality monitor$" or "Quality metric" or "Quality criter$" or "Performance indicator" or "Process PRE/2 care" or "Performance measure" or "Benchmark" or "benchmarking" or "Outcome measure" or "Outcome indicator$" or "Consensus" or "delphi") AND TITLE-ABS-KEY ("Primary PRE/2 care" or "General PRE/1 practi$" or "general practice" or "general practitioners" or "Clinical practice" or "Family practice" or "primary PRE/2 physician" or "healthcare delivery") AND TITLE-ABS-KEY ("primary PRE/2 prevention" OR "secondary PRE/2 prevention" OR "Prevention" OR "Control") AND ( EXCLUDE ( DOCTYPE,"cp" ) OR EXCLUDE ( DOCTYPE,"ed" ) ) AND ( LIMIT-TO ( PUBYEAR,2022) OR LIMIT-TO ( PUBYEAR,2021) OR LIMIT-TO ( PUBYEAR,2020) OR LIMIT-TO ( PUBYEAR,2019) OR LIMIT-TO ( PUBYEAR,2018) OR LIMIT-TO ( PUBYEAR,2017) OR LIMIT-TO ( PUBYEAR,2016) OR LIMIT-TO ( PUBYEAR,2015) OR LIMIT-TO ( PUBYEAR,2014) OR LIMIT-TO ( PUBYEAR,2013) OR LIMIT-TO ( PUBYEAR,2012) ) AND ( LIMIT-TO ( LANGUAGE,"English" ) ) |

**6) The authors indicated that they used the PRISMA checklist for systematic reviews, but the following aspects of the checklist were inadequately tackled: eligibility criteria (item #5); search strategy (item #7); and synthesis methods (item #13).**

**Response:** Thank you for this important comment. We have elaborated the sections on eligibility criteria, search strategy and synthesis methods. However, for the synthesis method, some of the criteria in the PRISMA were not applicable as we did not undertake meta-analysis so we have mentioned “NA” not applicable in S1 File PRISMA checklist.

**Response for eligibility criteria (item #5):**

To avoid confusion, we have now separated eligibility criteria with Population, Interventions, Comparator and Outcome (PICO) elements and elaborated further in Page 3, Lines 103-116, as copied below:

***Eligibility criteria***

We developed a broader eligibility criterion to include all potential articles with sufficient details on the quality indicators for primary prevention of CVD. All study designs, except case report, case series, letter to editor, protocol, conference abstracts, were included. We included full-text articles published in English language with sufficient details on the definition of quality indicators. Further details on eligibility criteria are described according to Population, Interventions, Comparator and Outcome (PICO) elements:

- **Population:** People without CVD being assessed, managed or treated in the primary care setting for primary prevention of CVD.
- **Interventions:** Development, validation, implementation, and evaluation of quality indicators targeting the management of risk factors for CVD.
- **Comparator:** Condition, type of quality indicator, themes, domains of primary care services.
- **Outcome:** Quality indicators in for primary prevention of CVD.

**Response for search strategy (item #7):**

We have now elaborated the section in the search strategy and have provided more detail related to search strategy in Page 3-4, Lines 125-134, as copied below:

The search terms used were “cardiovascular disease” or “stroke” AND “risk factors” AND “quality indicators” AND “primary care” AND “prevention”. Boolean operators such as AND, and OR were used to combine these search terms. The search terms were identified based on the initial scoping searches of the literature in Google Scholar and refined based on five relevant articles. Truncation (e.g., cardiovasc*, cerebrovasc*, stroke*) and Wildcards (e.g., cardiovasc$, cerebrovasc$) were used to broaden results and capture variations of words. We searched each database using a range of Medical Subject Headings (MeSH) terms to find all potentially relevant articles. The summary of the different databases and full search strategy is documented in **S1 Table**. as copied below

**S1 Table.** Overview of search strategy

| Database | Search strategy |
| --- | --- |
| Ovid Medline | 1 (Cardiovasc* adj1 disease*).tw.  2 (heart adj1 disease*).tw.  3 myocardial infarction/  4 Cerebrovasc* event*.tw.  5 Cerebrovasc* disease*.tw.  6 Cerebrovasc* accident*.tw.  7 (Cardiac adj1 failure).tw.  8 Stroke*.tw. or stroke/  9 Transient isch*.tw.  10 TIA.tw.  11 (Haemorrhag* adj1 stroke).tw.  12 (hemorrhag* adj1 stroke).tw.  13 1 or 2 or 3 or 4 or 5 or 6 or 7 or 8 or 9 or 10 or 11 or 12  14 Risk*.tw.  15 Hypertension.tw.  16 (High adj2 pressure).tw.  17 Dyslipi*.tw.  18 Hyperlipi*.tw.  19 Smoking.tw.  20 atrial fibrillation.tw.  21 Diabetes.tw. or diabetes mellitus/  22 Alcohol.tw.  23 (Physical adj2 activity).tw.  24 Obesity.tw.  25 ((Mental adj1 health) or (mental adj1 disorder*) or depress* or anxiety or psychiat* or psycho* or well-being or (quality adj1 life) or (self adj1 esteem) or (self adj1 perception)).tw.  26 14 or 15 or 16 or 17 or 18 or 19 or 20 or 21 or 22 or 23 or 24 or 25  27 (primary adj2 prevention).tw.  28 (secondary adj2 prevention).tw.  29 (Prevention or control).tw.  30 27 or 28 or 29  31 (Quality adj1 care*).tw.  32 Quality assessment.tw.  33 Quality indicator*.tw.  34 Quality assurance.tw.  35 (medication* or medicine*).tw.  36 Quality improvement.tw.  37 Quality tool*.tw.  38 Quality monitor*.tw.  39 Quality metric.tw.  40 Quality criter*.tw.  41 Performance indicator.tw.  42 (Process adj2 care).tw.  43 Performance measure.tw.  44 Benchmark.tw. or benchmarking/  45 Outcome measure.tw.  46 Outcome indicator*.tw.  47 (Consensus or delphi).tw.  48 31 or 32 or 33 or 34 or 35 or 36 or 37 or 38 or 39 or 41 or 42 or 43 or 44 or 45 or 46 or 47  49 (Primary adj2 care).tw.  50 (General adj1 practi*).tw. or exp general practice/ or general practitioners/  51 Clinical practice.tw.  52 Family practice.tw.  53 (primary adj2 physician).tw.  54 healthcare delivery.tw.  55 49 or 50 or 51 or 52 or 53 or 54  56 13 and 26 and 30 and 48 and 55  57 limit 56 to (english language and yr="2012 -Current") |
| Ovid EMBASE | 1 (Cardiovasc* adj1 disease*).tw.  2 (heart adj1 disease*).tw.  3 myocardial infarction/  4 Cerebrovasc* event*.tw.  5 Cerebrovasc* disease*.tw.  6 Cerebrovasc* accident*.tw.  7 (Cardiac adj1 failure).tw.  8 Stroke*.tw. or stroke/  9 Transient isch*.tw.  10 TIA.tw.  11 (Haemorrhag* adj1 stroke).tw.  12 (hemorrhag* adj1 stroke).tw.  13 1 or 2 or 3 or 4 or 5 or 6 or 7 or 8 or 9 or 10 or 11 or 12  14 Risk*.tw.  15 Hypertension.tw.  16 (High adj2 pressure).tw.  17 Dyslipi*.tw.  18 Hyperlipi*.tw.  19 Smoking.tw.  20 atrial fibrillation.tw.  21 Diabetes.tw. or diabetes mellitus/  22 Alcohol.tw.  23 (Physical adj2 activity).tw.  24 Obesity.tw.  25 ((Mental adj1 health) or (mental adj1 disorder*) or depress* or anxiety or psychiat* or psycho* or well-being or (quality adj1 life) or (self adj1 esteem) or (self adj1 perception)).tw.  26 14 or 15 or 16 or 17 or 18 or 19 or 20 or 21 or 22 or 23 or 24 or 25  27 (primary adj2 prevention).tw.  28 (secondary adj2 prevention).tw.  29 (Prevention or control).tw.  30 27 or 28 or 29  31 (Quality adj1 care*).tw.  32 Quality assessment.tw.  33 Quality indicator*.tw.  34 Quality assurance.tw.  35 (medication* or medicine*).tw.  36 Quality improvement.tw.  37 Quality tool*.tw.  38 Quality monitor*.tw.  39 Quality metric.tw.  40 Quality criter*.tw.  41 Performance indicator.tw.  42 (Process adj2 care).tw.  43 Performance measure.tw.  44 Benchmark.tw. or benchmarking/  45 Outcome measure.tw.  46 Outcome indicator*.tw.  47 (Consensus or delphi).tw.  48 31 or 32 or 33 or 34 or 35 or 36 or 37 or 38 or 39 or 41 or 42 or 43 or 44 or 45 or 46 or 47  49 (Primary adj2 care).tw.  50 (General adj1 practi*).tw. or exp general practice/ or general practitioners/  51 Clinical practice.tw.  52 Family practice.tw.  53 (primary adj2 physician).tw.  54 healthcare delivery.tw.  55 49 or 50 or 51 or 52 or 53 or 54  56 13 and 26 and 30 and 48 and 55  57 limit 56 to (english language and yr="2012 -Current")  58 limit 57 to conference abstracts  59 57 not 58 |
| CINAHL Plus | (Cardiovasc* adj1 disease* OR heart adj1 disease* OR Stroke* OR Transient isch*or Haemorrhag* adj1 stroke OR hemorrhag* adj1 stroke OR TIA OR myocardial infarction OR Cerebrovasc* event* OR Cerebrovasc* disease* OR Cerebrovasc* accident* OR cardiac failure) AND (Risk* OR Hypertension OR High adj2 pressure OR Dyslipi* OR Hyperlipi* OR atrial adj1 fibrillation OR Smoking OR Diabetes OR diabetes mellitus OR Alcohol OR Physical adj2 activity OR Obesity OR Mental health OR mental disorder* OR depress* OR anxiety OR psychiat* OR well-being OR quality of life OR self esteem OR self perception) AND (Quality adj1 care* OR Quality assessment OR Quality indicator* OR Quality assurance OR medication* OR medicine* Quality improvement OR Quality tool* OR Quality monitor* OR Quality metric OR Quality criter* OR Performance indicator OR Process adj2 care OR Performance measure OR Benchmark OR benchmarking OR Outcome measure OR Outcome indicator* OR Consensus OR delphi) AND (Primary adj2 care OR General adj1 practi* OR general practice OR general practitioners OR Clinical practice OR Family practice OR primary adj2 physician OR healthcare delivery) AND (primary adj2 prevention OR secondary adj2 prevention OR prevention OR control) |
| SCOPUS | TITLE-ABS-KEY ("cardiovasc$ PRE/1 disease$" OR "heart PRE/1 disease$" OR "myocardial infarction" OR "cerebrovasc$ event$" OR "cerebrovasc$ disease$" OR "cerebrovasc$ accident$" OR "cardiac failure") OR ("stroke" OR "transient isch$" OR "haemorrhag$ PRE/1 stroke" OR "hemorrhag$ PRE/1 stroke") AND TITLE-ABS-KEY ("Risk$" OR "Hypertension" OR "High PRE/2 pressure" OR "Dyslipi$" OR "Hyperlipi$" OR "Smoking" OR "Diabetes" OR "diabetes mellitus" OR "Alcohol" OR "Physical PRE/2 activity" OR "Obesit$" OR "mental health" OR "mental disorder" OR "depress$" OR "anxiety" OR "psycho$" OR "psychiat$" OR "well-being" OR "quality of life" OR "self esteem" OR "self perception") AND TITLE-ABS-KEY ("Quality PRE/1 care$" or "Quality assessment" or "Quality indicator$" or "Quality assurance" or "medication$" or "medicine$" "Quality improvement" or "Quality tool$" or "Quality monitor$" or "Quality metric" or "Quality criter$" or "Performance indicator" or "Process PRE/2 care" or "Performance measure" or "Benchmark" or "benchmarking" or "Outcome measure" or "Outcome indicator$" or "Consensus" or "delphi") AND TITLE-ABS-KEY ("Primary PRE/2 care" or "General PRE/1 practi$" or "general practice" or "general practitioners" or "Clinical practice" or "Family practice" or "primary PRE/2 physician" or "healthcare delivery") AND TITLE-ABS-KEY ("primary PRE/2 prevention" OR "secondary PRE/2 prevention" OR "Prevention" OR "Control") AND ( EXCLUDE ( DOCTYPE,"cp" ) OR EXCLUDE ( DOCTYPE,"ed" ) ) AND ( LIMIT-TO ( PUBYEAR,2022) OR LIMIT-TO ( PUBYEAR,2021) OR LIMIT-TO ( PUBYEAR,2020) OR LIMIT-TO ( PUBYEAR,2019) OR LIMIT-TO ( PUBYEAR,2018) OR LIMIT-TO ( PUBYEAR,2017) OR LIMIT-TO ( PUBYEAR,2016) OR LIMIT-TO ( PUBYEAR,2015) OR LIMIT-TO ( PUBYEAR,2014) OR LIMIT-TO ( PUBYEAR,2013) OR LIMIT-TO ( PUBYEAR,2012) ) AND ( LIMIT-TO ( LANGUAGE,"English" ) ) |

**Response for synthesis methods (item #13):** We have now provided more detail in the “Data extraction and synthesis” section in Page 5, Lines 171-196, as copied below:

## *Data extraction and synthesis*

The excel file template used for data extraction was initially developed by KB, MFK, MTO, and DAC, with inputs from the advisory group (other co-authors), and modified as the extraction progressed. The final data extraction table for study characteristics included information for author, year of publication, country, study design/methodology, and study period **(S4 Table)**. Other important information collected on summary of quality indicators include condition, domains, theme, type of indicator, age group (years), numerator, denominator, and the frequency of collection **(S5 Table)**. Data extraction was undertaken by KB, reviewed by MTO, and any disagreements were adjudicated by MFK or DAC.

Details about the quality indicators including numerator, denominator, age group (in years) and frequency of collection were extracted and summarised in tabular format **(S5 Table)**. For further data synthesis, themes and conditions were identified based on literature [14, 17-21], pre-tested across the initial set of the articles. For categorisation of domains and type of indicator, a theory informed conceptual framework **(Fig 2)** was developed from existing literature [14, 18, 20-23], and the Donabedian’s multidimensional framework [24, 25] of structure, process and outcome was used. This framework facilitated a comprehensive summary and evaluation of care quality across domains of primary care services [26], as described below.

## a) **Structure indicators** include infrastructure or human resources-related indicators that designate the conditions under which primary care is provided (*organisation of care*);

## b) **Process indicators** include the actions and processes of clinical care delivered by primary care physician, such as screening, ordering diagnostic tests, prescribing treatment i.e. medications, or monitoring (*assessment of metabolic risk factors, global risk assessment, lifestyle management, prescription of medications, risk communication/advice, or referral*); and

## c) **Outcome indicators** reflect changes in individuals resulting from the intervention provided at the primary care (*attainment of risk factor targets*).

We have now separated data analysis section, section in Page 6, Lines 225-231, as copied below:

***Data analysis***

Descriptive statistics (frequencies and percentages) were used to summarise results using Microsoft Excel’s advanced analysis features, particularly by pivot tables. Data were presented in tabular format with narrative description given the nature of the review. Figures, where applicable, were developed to visualise the flow diagram, methodological quality heat map, and distribution of quality indicators using Microsoft Excel or Microsoft PowerPoint graph features.

**7) How did the authors find the grey literature? It appears that such grey literature were pulled from sources that were outside the four stated databases. Please clarify.**

**Response:** Thank you for this comment. We have added the following sentences to clarify the sources of grey literature in Page 4, Lines 152-157, as copied below:

We searched grey literature in the form of unpublished empirical research papers and databases on quality indicators primarily from countries with similar, well-established primary care systems, such as the United Kingdom (UK), Canada and Australia. Grey literature was searched using google scholar and websites of respective government or non-government organisations (e.g., National Institute for Health and Clinical Excellence Australian Institute of Health and Welfare). The list of organisations researched are documented in **S2 Table**, as copied below:

| **Organisations** | **Webpage** |
| --- | --- |
| Australian Council on Healthcare Standards | <https://www.achs.org.au/> |
| The Australian Commission on Safety and Quality in Health Care List | <https://www.safetyandquality.gov.au/standards/nsqhs-standards> |
| Australian Institute of Health and Welfare | <https://www.aihw.gov.au/> |
| Australian Government of Health and Ageing | <https://www.health.gov.au/> |
| Clinical Information Access Programme | <https://www.ciap.health.nsw.gov.au/> |
| National Institute of Clinical Studies | <https://catalogue.nla.gov.au/catalog/3625531> |
| Victorian Government Health Information | <https://www.health.vic.gov.au/> |
| Victorian Government Public Sector Residential Aged Care Services (PSRACS) quality Indicators | <https://www.health.vic.gov.au/residential-aged-care/quality-indicators-in-public-sector-residential-aged-care-services> |
| Improvement Foundation | <https://www.acnc.gov.au/charity/charities/721fd63e-38af-e811-a960-000d3ad24282/people> |
| Royal Australian College of General Practitioners | <https://www.racgp.org.au/> |
| Bettering the Evaluation and Care of Health (BEACH) studies | <https://www.sydney.edu.au/medicine-health/our-research/research-centres/bettering-the-evaluation-and-care-of-health.html> |
| Quality indicators. The Royal Australian College of General Practitioners: | <https://www.racgp.org.au/> |
| National Aged Care Mandatory Quality Indicator Program | <https://www.health.gov.au/our-work/qi-program> |
| Quality indicator app | <https://www.qpsbenchmarking.com/solutions/national-quality-indicator-app/> |
| Clinical Indicators | <https://www.racgp.org.au/running-a-practice/practicemanagement/general-practice-governance/clinical-indicators> |
| Care Quality Commission | <https://www.cqc.org.uk/> |
| CQUIN – Commissioning for Quality and Innovation Indicator | <https://www.england.nhs.uk/nhs-standard-contract/cquin/> |
| Department of Health | <https://www.gov.uk/government/organisations/department-of-health-and-social-care> |
| Dr. Foster Intelligence | <https://telstrahealth.co.uk/product/healthcare-intelligence-portal-quality/> |
| The Information Centre | <https://www.gov.uk/government/organisations/nhs-information-centre> |
| Clinical and Health Outcomes Knowledge Base | <https://digital.nhs.uk/article/1885/compendium-of-population-health-indicators> |
| NHS North West | <https://www.england.nhs.uk/north-west/> |
| National Institute for Health and Clinical Excellence (NICE) | <https://www.nice.org.uk/> |
| The Health Foundation | <https://www.health.org.uk/> |
| Stroke quality indicator | <https://archive.uhb.nhs.uk/stroke-quality-indicator.htm> |
| Accreditation Canada | <https://accreditation.ca/> |
| Canadian Institute for Health Information (CIHI) | <https://www.cihi.ca/en> |
| Health Canada | <https://www.canada.ca/en/health-canada.html> |
| Health Council of Canada | <https://www.healthcouncilcanada.ca/> |
| Public Health Agency of Canada | <https://www.canada.ca/en/public-health.html> |

**8) The rationale of the study is towards stroke prevention, however, the authors barely touched on this during the discussion. The authors should consider discussing the implications of the findings, particularly the finding that most of the indicators focused on assessment risk factors at the expense of structure and outcome indicators, on stroke prevention. Could it be that the inadequacy of structure and outcome indicators undercut the stroke prevention measures? If so, how?**

**Response 1:** We have now highlighted the implication of our findings with a focus on structural and outcome indicators in the “Discussion” section in Page 12-13, Lines 441-443 and 448-457, as copied below. More importantly, we have considered cardiovascular disease as a broader term that includes both heart disease and stroke. The focus of this systematic review is both, heart disease and stroke, as they share common risk factors.

Outcome indicators are also important to enable monitoring the overall result of the course of action or treatments provided, than a single aspect of care [17]. However, monitoring of the structure indicators and its outcome is often neglected in primary care [102]. This could be because structural interventions are considered less important for the primary prevention of CVD (e.g. stroke) [103]. A combination and balance of structure, process and outcome quality indicators aligned with the Donabedian framework is important for measuring the quality of care for CVD prevention in primary care settings [24]. The inadequacy of structure and outcome indicators can impede CVD and stroke prevention measures as this may lead to inadequate monitoring of the patient outcomes, infrastructure support and accountability [104]. These indicators are critical to deliver high- quality care especially targeting monitoring and following up those with sub-optimal outcomes [105]. There is a need for the right mix of the indicators, including structure and outcome indicators, to identify the gaps in workforce or infrastructure, ensure quality for primary prevention, and monitor the patient outcomes [106]. Importantly, short term (repeated measures of the lifestyle risk factors), intermediate outcome indicators such as control of risk factors (e.g., attaining blood pressure, blood glucose, smoking cessation targets) are needed for each risk factors for CVD prevention in primary care settings.

We have further highlighted the need for developing and monitoring lifestyle advice related indicators for primary prevention of CVD in the “Discussion” section in Page 12, Lines 413-419, as copied below.

Lifestyle advice is a critical element of care for the primary prevention of CVD and inadequate indicators could lead to low monitoring and follow-up [91]. In a study with American adults, authors reported that lifestyle advice from their general practitioners led to behavioural changes, such as reduction in weight or increased physical activity, thus, improving primary prevention of CVD [92]. This low reporting of lifestyle advice could be potentially due to incomplete records of assessment or advice on lifestyle risk factors [93-95].

**B) Reviewer #2:**

I accept manuscript which discuss the quality indicators for primary CVD prevention Quality indicators for the primary prevention of cardiovascular disease in primary care A systematic review for publication

**Response:** We thank the reviewer for the positive feedback on the work presented.
